# High‐Performance Orange–Red Organic Light‐Emitting Diodes with External Quantum Efficiencies Reaching 33.5% based on Carbonyl‐Containing Delayed Fluorescence Molecules

**DOI:** 10.1002/advs.202104435

**Published:** 2021-12-19

**Authors:** Ruming Jiang, Xing Wu, Hao Liu, Jingjing Guo, Dijia Zou, Zujin Zhao, Ben Zhong Tang

**Affiliations:** ^1^ State Key Laboratory of Luminescent Materials and Devices Guangdong Provincial Key Laboratory of Luminescence from Molecular Aggregates South China University of Technology Guangzhou 510640 China; ^2^ Shenzhen Institute of Aggregate Science and Technology School of Science and Engineering The Chinese University of Hong Kong Shenzhen Guangdong 518172 China; ^3^ AIE Institute Guangzhou Development District Huangpu Guangzhou 510530 China

**Keywords:** horizontal dipole orientation, orange–red emitters, organic light‐emitting diodes, spin–orbit coupling, thermally activated delayed fluorescence

## Abstract

Developing orange to red purely organic luminescent materials having external quantum efficiencies (*η*_ext_s) exceeding 30% is challenging because it generally requires strong intramolecular charge transfer, efficient reverse intersystem crossing (RISC), high photoluminescence quantum yield (*Φ*_PL_), and large optical outcoupling efficiency (*Φ*_out_) simultaneously. Herein, by introducing benzoyl to dibenzo[*a*,*c*]phenazine acceptor, a stronger electron acceptor, dibenzo[*a*,*c*]phenazin‐11‐yl(phenyl)methanone, is created and employed for constructing orange–red delayed fluorescence molecules with various acridine‐based electron donors. The incorporation of benzoyl leads to red‐shifted photoluminescence with accelerated RISC, reduced delayed lifetimes, and increased *Φ*_PL_s, and the adoption of spiro‐structured acridine donors promotes horizontal dipole orientation and thus renders high *Φ*_out_s. Consequently, the state‐of‐the‐art orange–red organic light‐emitting diodes are achieved, providing record‐high electroluminescence (EL) efficiencies of 33.5%, 95.3 cd A^−1^, and 93.5 lm W^‒1^. By referring the control molecule without benzoyl, it is demonstrated that the presence of benzoyl can exert significant positive effect over improving delayed fluorescence and enhancing EL efficiencies, which can be a feasible design for robust organic luminescent materials.

## Graphical abstract

High‐efficiency orange to red delayed fluorescence molecules are obtained by integrating benzoyl‐bearing dibenzo[*a*,*c*]phenazine acceptor and spiro‐structured acridine donors, rendering superb electroluminescence performance with record‐beating external quantum, current, and power efficiencies of 33.5%, 95.3 cd A^−1^, and 93.5 lm W^−1^, respectively.

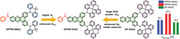

## 1 Introduction

Organic light‐emitting diodes (OLEDs)^[^ ^1^, ^2^ ^]^ have received continuous and intense research interests of academia and industry owing to their distinct merits that ensue promising applications in display and lighting devices. Maximizing device efficiencies, minimizing energy consumption, and reducing manufacturing cost are the fundamental prerequisites for massive commercialization of OLEDs,^[^ ^3^ ^]^ which greatly rely on the exploration of robust light‐emitting materials. Purely organic thermally activated delayed fluorescence (TADF) materials without any precious noble metals are currently in the spotlight because they are not only low‐cost but also able to harvest both singlet and triplet excitons via reverse intersystem crossing (RISC) to boost electroluminescence (EL) efficiencies.^[^ ^4^, ^5^, ^6^, ^7^ ^]^ Constructing highly twisted electron‐donor and acceptor (D‐A) structures can bring about adequate separation of the highest occupied molecular orbital (HOMO) and the lowest unoccupied molecular orbital (LUMO), making energy splitting (∆*E*_ST_) between the lowest singlet excited (S_1_) state and the lowest triplet excited (T_1_) state reduced to ensure rapid RISC and thus improve exciton utilization.^[^ ^8^, ^9^, ^10^, ^11^, ^12^ ^]^

After a landmark breakthrough by Adachi's group, many efficient sky‐blue, green, and yellow TADF materials have been developed thus far and their corresponding OLEDs have reached the highest external quantum efficiencies (*η*_ext_s) of around 38%.^[^ ^13^, ^14^, ^15^, ^16^ ^]^ In contrast, the device efficiencies of orange‐to‐red TADF materials with EL peaks over 560 nm remain far behind.^[^ ^17^, ^18^, ^19^, ^20^, ^21^ ^]^ In order to realize efficient long‐wavelength EL emissions, strengthening intramolecular charge transfer (ICT) effect in a highly twisted D‐A framework is widely used in the design of TADF materials.^[^ ^22^, ^23^, ^24^, ^25^ ^]^ But according to the Franck–Condon transition principle,^[^ ^26^ ^]^ the separation of HOMOs and LUMOs as well as strong ICT effect often leads to low radiative decay rate and weak oscillator strength, thereby resulting in decreased photoluminescence (PL) quantum yields (*Φ*_PL_s). On the other hand, the horizontal dipole ratio (Θ_//_) of the molecule should also be enhanced to break the limit of 20‒30% optical outcoupling efficiency (*Φ*_out_).^[^ ^27^, ^28^ ^]^ In fact, some orange–red TADF materials obtained by great efforts possess small Δ*E*_ST_s and high *Φ*_PL_s, but their EL efficiencies are less than satisfactory due to their low Θ_//_s, and vice versa.^[^ ^29^, ^30^, ^31^, ^32^, ^33^ ^]^ It is virtually challenging to simultaneously achieve a small ∆*E*_ST_, a high *Φ*_PL_, and a large *Φ*_out_, especially for TADF materials with long‐wavelength emissions. In consequence, there are only very limited reports on the efficient orange–red TADF OLEDs with highest *η*_ext_s of around 30%.^[^ ^3^, ^34^, ^35^, ^36^, ^37^ ^]^

To break through the predicament, in this contribution, we propose a feasible molecular design for orange–red TADF materials and synthesize a series of tailor‐made luminescent molecules DPPM‐DMAC, DPPM‐DPAC, DPPM‐SFAC, DPPM‐STAC, and DPPM‐SXAC. In these molecules, various acridine derivatives 9,9‐dimethyl‐9,10‐dihydroacridine (DMAC), 9,9‐diphenyl‐9,10‐dihydroacridine (DPAC), spiro[acridine‐9,9'‐fluorene] (SFAC), spiro[acridine‐9,9'‐thioxanthene] (STAC), and spiro[acridine‐9,9'‐xanthene] (SXAC), are adopted as electron donors, in which the substituents at the 9‐position of acridine are altered to optimize Θ_//_s. A large electron acceptor of dibenzo[*a*,*c*]phenazin‐11‐yl(phenyl)methanone (DPPM) is created by grafting benzoyl to dibenzo[*a*,*c*]phenazine (DP) to increase electron‐withdrawing ability for generating longer‐wavelength emissions. The presence of carbonyl group is also anticipated to enhance spin–orbit coupling (SOC) matrix elements^[^ ^38^, ^39^, ^40^ ^]^ to facilitate RISC and benefit delayed fluorescence. For comparison, a control TADF molecule (DP‐SXAC) containing a DP acceptor and SXAC donor is prepared and studied. The systematical experiments and theoretical calculations reveal that these DPPM‐based molecules exhibit more efficient delayed fluorescence with longer emission wavelengths, higher *Φ*_PL_s, and shorter delayed lifetimes than DP‐SXAC. The OLEDs employing DPPM‐based molecules as emitters radiate orange–red lights and achieve a record‐beating *η*_ext_ of 33.5%, much superior to that of DP‐SXAC‐based device (19.5%). The high *Φ*_PL_s, efficient RISC as well as large *η*_out_s account for the outstanding EL performances of these DPPM‐based molecules.

## 2 Result and Discussion

**Figure 1:**
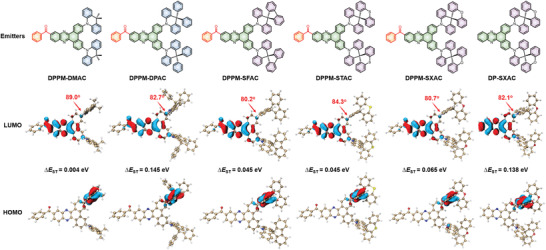
Molecular structures, dihedral angles between donor and acceptor in optimized ground geometry, frontier molecular orbital distributions, and the calculated Δ*E*_ST_ values of DPPM‐DMAC, DPPM‐DPAC, DPPM‐SFAC, DPPM‐STAC, DPPM‐SXAC, and DP‐SXAC.

The molecular structures of DPPM‐based molecules (DPPM‐DMAC, DPPM‐DPAC, DPPM‐SFAC, DPPM‐STAC, DPPM‐SXAC), and the control molecule DP‐SXAC without benzoyl are shown in **Figure** **1**, and their detailed synthetic routes involving two steps are shown in Schemes S1 and S2 of the Supporting Information. Briefly, the key precursors DP‐Br and DPPM‐Br are synthesized by cyclization reactions of 3,6‐dibromo‐phenanthrenequinone with *o*‐phenylenediamine and (3,4‐diaminophenyl)phenylmethanone, respectively, which are coupled with acridine‐based donors via Buchwald–Hartwig C–N coupling reaction to produce target molecules in good yields. They are fully characterized by ^1^H NMR, ^13^C NMR, and high‐resolution mass spectrometry, and the results are in good agreement with the molecular structures. The detailed synthetic procedures and characterization data are described in the Supporting Information.

The thermal stability of these molecules is examined by thermogravimetric analysis. As shown in Figure S1 in the Supporting Information, they have excellent thermal stability with high decomposition temperatures (*T*_d_s, corresponding to 5% loss of initial weight) of 447 °C for DPPM‐DMAC, 493 °C for DPPM‐DPAC, 532 °C for DPPM‐SFAC, 542 °C for DPPM‐STAC, and 525 °C for DPPM‐SXAC. The *T*_d_s of DPPM‐SFAC, DPPM‐STAC, and DPPM‐SXAC are obviously higher than those of DPPM‐DMAC and DPPM‐DPAC, indicating the spiro structure has positive effect on increasing thermal stability. Besides, the *T*_d_ of DPPM‐SXAC is also much higher than that of DP‐SXAC (497 °C), revealing that the introduction of benzoyl has improved the thermal stability of the molecule. On the other side, all of these molecules hold good electrochemical stability as well, as evidenced by the reversible oxidation and reduction processes in cyclic voltammetry measurement (Figure S2, Supporting Information). According to the thresholds of the first oxidation and reduction waves, the HOMO and LUMO energy levels are calculated to be ‒5.28 and ‒3.46 eV for DPPM‐DMAC, ‒5.42 and ‒3.55 eV for DPPM‐DPAC, ‒5.36 and ‒3.51 eV for DPPM‐SFAC, ‒5.41 and ‒3.51 eV for DPPM‐STAC, ‒5.41 and ‒3.52 eV for DPPM‐SXAC, and ‒5.41 and ‒3.24 eV for DP‐SXAC. Compared with other molecules, DPPM‐DMAC has a higher HOMO energy level owing to the stronger electron‐donating ability of DMAC.^[^ ^3^, ^41^ ^]^ And the DPPM‐based molecules exhibit lower LUMO energy levels than DP‐SXAC, which is ascribed to the stronger electron‐withdrawing ability of DPPM acceptor resulting from the additional carbonyl group.

To gain insights into the electronic structures, density functional theory calculation is carried out to simulate the optimized geometries at the ground state and frontier molecular orbital distributions. All the molecules adopt highly twisted geometries with large torsional angles over 80^o^ between donors and acceptors, facilitating the separation of HOMOs and LUMOs (Figure 1). The LUMOs are predominantly concentrated on DP and carbonyl groups, while the HOMOs are mainly located on acridine moieties, validating the presence of ICT characteristics. The Δ*E*_ST_s are calculated to be 0.004 eV for DPPM‐DMAC, 0.145 eV for DPPM‐DPAC, 0.045 eV for DPPM‐SFAC, 0.045 eV for DPPM‐STAC, and 0.065 eV for DPPM‐SXAC, ensuring efficient RISC and thus delay fluorescence. Due to the electron‐withdrawing effect of carbonyl group, the HOMO and LUMO distributions become more separate in DPPM‐SXAC than in DP‐SXAC, as evidenced by the less electron cloud locating on the phenyl ring connected with acridine moiety, resulting in a much smaller Δ*E*_ST_ (0.065 eV) of DPPM‐SXAC than that of DP‐SXAC (0.138 eV).

**Figure 2:**
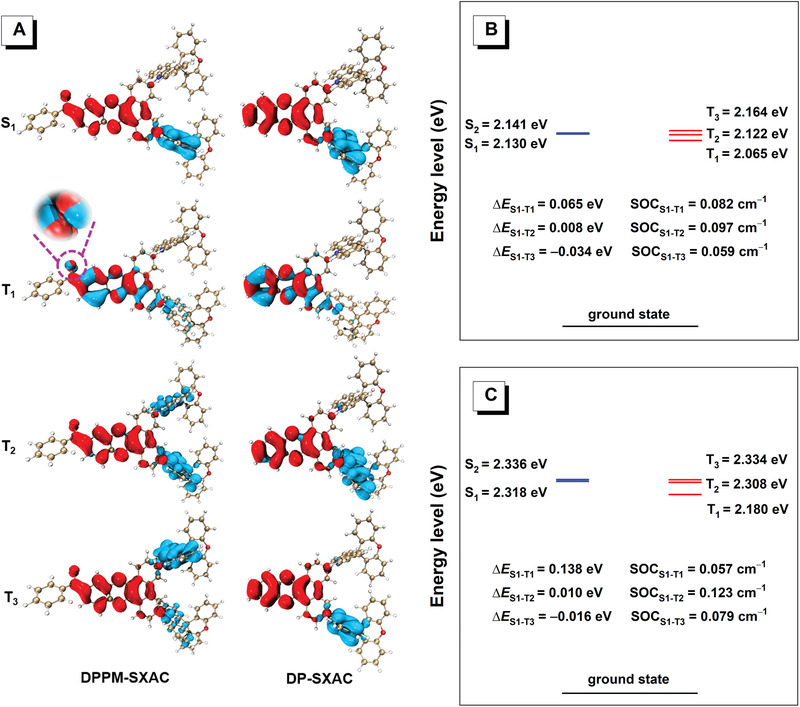
A) Hole (red) and electron (blue) analyses of excited singlet and triplet states of DPPM‐SXAC and DP‐SXAC with the spin–orbit coupling (SOC) matrix elements. The encircled and enlarged parts indicate the n orbital character. Calculated energy levels, Δ*E*_ST_ values, and SOC matrix elements of excited singlet and triplet states of B) DPPM‐SXAC and C) DP‐SXAC.

Moreover, the analysis on the hole and electron distributions of the excited states discloses that, for the S_1_ state, the hole and electron are distributed separately on the donor and acceptor, respectively, indicative of the typical charge transfer (CT) state dominated by *π*‒π* transition (**Figure** **2**A). Different from the S_1_ state, both hole and electron of the T_1_ state are primarily centered on the acceptor, implying the predominant characteristic of localized excitation state governed by n‒π* and *π*‒π* transition. The distinct difference in transition nature between S_1_ and T_1_ states is beneficial for the occurrence of RISC processes, rendering increased RISC rate.^[^ ^22^, ^42^, ^43^, ^44^ ^]^ In contrast, the second triplet excited (T_2_) and third triplet excited (T_3_) states of both molecules are dominated by the CT characters. Theoretically, up‐conversion of CT‐featured triplet states to CT‐featured singlet states is difficult.^[^ ^22^ ^]^ But T_2_ and T_3_ lie close to S_1_, with small energy level splitting and large SOC matrix elements between S_1_ and T_2_, and S_1_ and T_3_ (Figure 2B,C), suggesting these highly lying triplet excited states may contribute to spin‐flip event as well.^[^ ^45^, ^46^ ^]^ Interestingly, the calculated SOC matrix element between S_1_ and T_1_ of DPPM‐SXAC (0.082 cm^‒1^) is larger than that of DP‐SXAC (0.057 cm^‒1^), benefiting from n‒π* transition of carbonyl group.^[^ ^38^, ^39^, ^40^ ^]^ These results demonstrate the introduction of benzoyl can exert apparent positive effect on RISC by not only reducing Δ*E*_ST_ but also enlarging SOC matrix element, leading to efficient delayed fluorescence with short delayed lifetimes. The promoted RISC is also favorable to suppress nonradiative decay of triplet excited states, and thus conducive to increasing PL efficiency.

**Figure 3:**
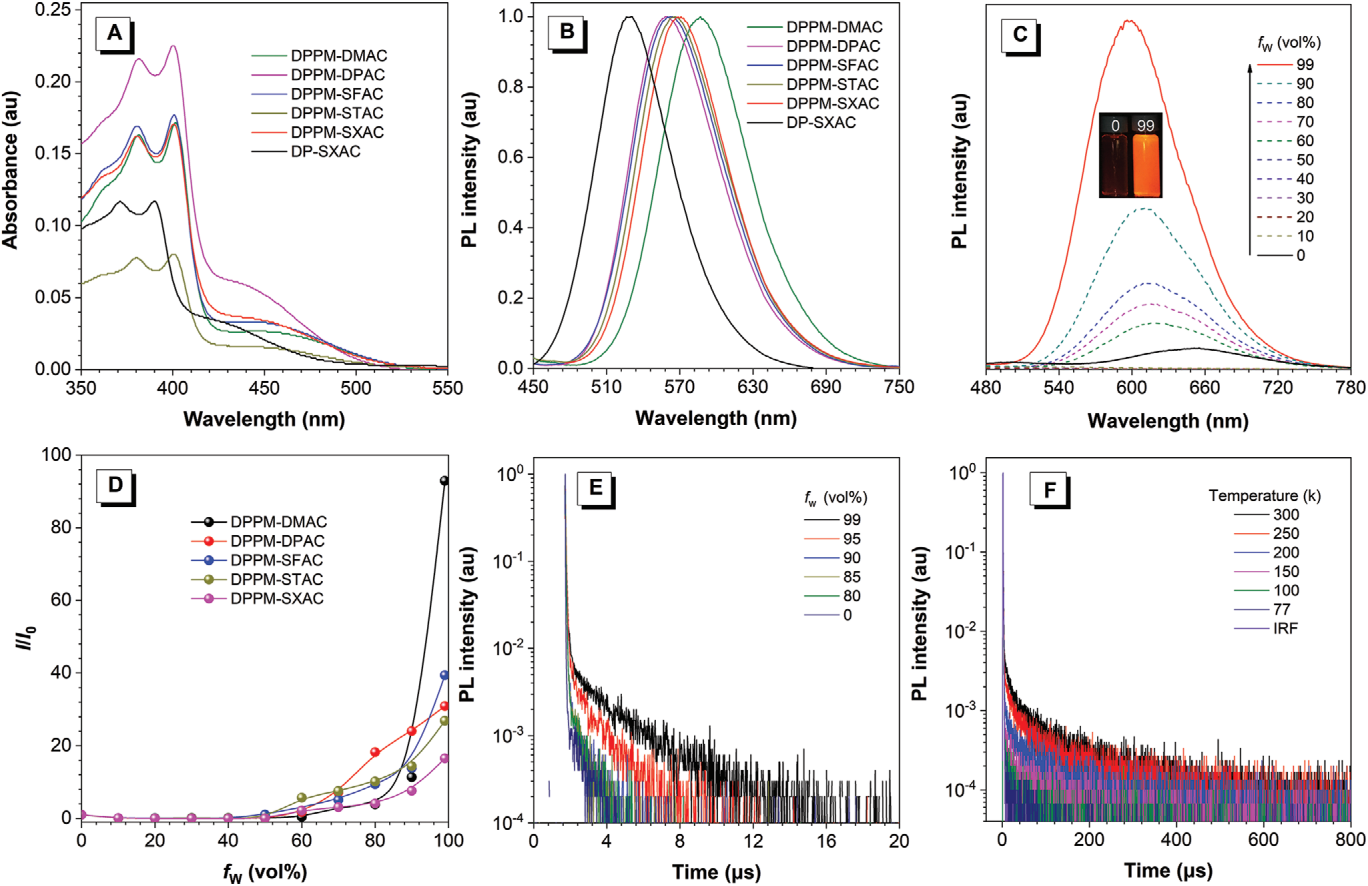
A) Absorption spectra of the new molecules in THF solutions (10^‒5^ m). B) PL spectra in doped films. C) PL spectra of DPPM‐SXAC in THF/water mixtures with different water fractions (*f*_w_; 10^‒5^ m). Inset: photos of DPPM‐SXAC in THF/water mixtures (*f*_w_ = 0 and 99%), taken under 365 nm excitation of a UV lamp. D) Plots of PL peak intensity versus *f*_w_, where *I*_0_ is the PL intensity in pure THF solution. E) Transient PL decay spectra of DPPM‐SXAC in THF/water mixtures with different *f*_w_s. F) Temperature‐dependent transient PL decay spectra of DPPM‐SXAC doped in CBP host.

DPPM‐based molecules represent analogous absorption spectral profiles in dilute tetrahydrofuran (THF) solutions (**Figure** **3**A). The intense absorption bands at around 400 nm are attributed to the *π*–π* transitions of the molecules, while the weak and broad absorption bands at about 450 nm are associated with ICT transitions from acridine‐based donors to DPPM acceptor. They show orange to red PL emissions (568‒603) with *Φ*_PL_s of 29‒44% in dilute toluene solutions, which are greatly red‐shifted to 655‒682 nm with decreased *Φ*_PL_s of 1‒4% in THF solutions (Figure S3 and Table S1, Supporting Information), due to strengthened ICT effect in polar solvent. On the contrary, DP‐SXAC shows an absorption band at about 430 nm in THF from ICT transition, and greatly blue‐shifted PL peaks at 532 in toluene and 598 nm in THF, due to its relatively weak ICT effect. On the other hand, the PL intensities in toluene can be apparently weakened by O_2_ (Figure S4, Supporting Information)_._ And the transient PL decay spectra of these molecules display obvious delayed components in O_2_‐free toluene bubbled with N_2_, but the delayed components are reduced greatly in the presence of O_2_ (Figure S5, Supporting Information). These findings suggest that the triplet excited states are involved in PL emissions.

In THF solutions, DPPM‐based molecules show faint PL emissions without distinct delayed fluorescence. By adding a poor solvent of water into their THF solutions, the PL peaks are progressively blue‐shifted and PL intensities are enhanced greatly (Figure 3C,D and Figure S6, Supporting Information). With the addition of a large amount of water, these luminogenic molecules form aggregates because of their hydrophobic nature, and the enhanced and blue‐shifted PL emissions in aggregates are caused by the lowered matrix polarity and restricted intramolecular motions.^[^ ^47^, ^48^, ^49^ ^]^ More interestingly, the PL lifetimes of these molecules get elongated apparently and the delayed components become more prominent at high water fractions, indicative of aggregation‐induced delayed fluorescence phenomenon (Figure 3E and Figure S7, Supporting Information), which is attributed to the promoted RISC by mainly impeding internal conversion of the excited states in condensed phase.^[^ ^9^ ^]^

DPPM‐based molecules show orange–red PL emissions with peaks at 557‒587 nm in doped films with 4,4′‐bis(carbazol‐9‐yl)biphenyl (CBP) host, while DP‐SXAC emits green PL emission peaking at 528 nm (Figure 3B). DPPM‐based molecules have similar *Φ*_PL_ values of 86‒94% in doped films, much higher than that of DP‐SXAC (60%). The promoted RISC can reduce nonradiative decay of triplet excited states and thus could be accountable for the higher *Φ*_PL_ of DPPM‐SXAC than DP‐SXAC. The ∆*E*_ST_s of DPPM‐based molecules are calculated to be 0.06‒0.25 eV from the onset of fluorescence and phosphorescence spectra at 77 K (Figure S8, Supporting Information), smaller than that of DP‐SXAC (0.34 eV). This finding is in good agreement with the calculation results. The transient PL decay spectra of these molecules in doped films display eminent double‐exponential decay with prompt and delayed components (**Table** **1** and Figure S9, Supporting Information). They possess better delayed fluorescence property than DP‐SXAC. The delayed fluorescence lifetime of DPPM‐SXAC (37 µs) is dramatically shorter than that of DP‐SXAC (1991 µs), validating the significant positive impact of carbonyl group on the acceleration of RISC. The temperature‐dependent transient PL decay spectra show that by increasing temperature from 77 to 300 K, the ratio of delayed fluorescence gradually rises (Figure 3F and Figure S10 and Table S2, Supporting Information). The triplet excited states undergo RISC to up‐convert to singlet excited states via thermal activation, verifying the TADF characteristics of these molecules.

**Table 1:** Photophysical properties, energy levels, and thermal stabilities of the new molecules

Molecule^a)^	*λ*_PL_ [nm]	*Φ*_PL_ [%]	*τ*_p_ [ns]	*τ*_d_ [µs]	*R*_d_ [%]	*k*_F_ [× 10^7^ s^‒1^]	*k*_IC_ [× 10^6^ s^‒1^]	*k*_ISC_ [× 10^7^ s^‒1^]	*k*_RISC_ [× 10^3^ s^‒1^]	∆*E*_ST_ [eV]	Θ_//_ [%]	HOMO/LUMO [eV]^b)^	*T*_d_ [°C]
DPPM‐DMAC	587	86	11	125	41	4.8	7.8	3.9	13.7	0.06	66	‒5.28/‒3.46	447
DPPM‐DPAC	557	87	8	455	35	7.3	11.0	4.6	3.4	0.25	69	‒5.42/‒3.55	493
DPPM‐SFAC	563	89	10	233	46	4.8	5.9	4.5	7.9	0.13	82	‒5.36/‒3.51	532
DPPM‐STAC	566	86	11	72	30	5.5	8.9	2.7	19.8	0.20	80	‐5.41/‐3.51	542
DPPM‐SXAC	570	94	12	37	19	6.2	3.9	1.5	33.4	0.17	82	‒5.41/‒3.52	525
DP‐SXAC	528	60	9	1991	69	2.1	1.4	7.7	1.6	0.34	79	‒5.41/‒3.24	497

^a)^ Abbreviations: *λ*_PL_ = PL peak; *Φ*_PL_ = absolute PL quantum yield, evaluated using an integrating sphere under nitrogen; *τ*_p_ and *τ*_d_ = lifetimes calculated from the prompt and delayed fluorescence decay, respectively; *R*_d_ = ratio of delayed component; *k*_F_ = fluorescence decay rate; *k*_IC_ = internal conversion rate from S_1_ to S_0_ states; *k*_ISC_ = intersystem crossing rate from S_1_ to T_1_ states; *k*_RISC_ = reverse intersystem crossing rate. ∆*E*_ST_ = energy splitting between S_1_ and T_1_ states, estimated from the high‐energy onsets of fluorescence and phosphorescence spectra at 77 K; Θ_//_ = horizontal dipole ratio. Photophysical data are measured from the doped films of the new molecules in CBP host (10 wt%) under nitrogen at 300 K.

^b)^ Measured by cyclic voltammetry in solutions.

**Figure 4:**
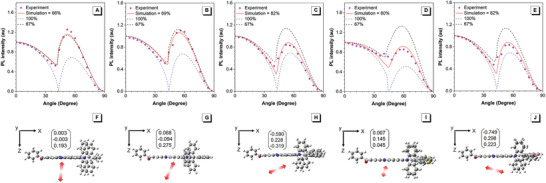
Measured (symbols) *p*‐polarized PL intensity (at PL peak wavelength) of the doped films of A) DPPM‐DMAC, B) DPPM‐DPAC, C) DPPM‐SFAC, D) DPPM‐STAC, and E) DPPM‐SXAC in CBP host (10 wt%). Simulated dipole moment vectors for F) DPPM‐DMAC, G) DPPM‐DPAC, H) DPPM‐SFAC, I) DPPM‐STAC, and J) DPPM‐SXAC in S_1_ state.

The Θ_//_s of these molecules are investigated by angle‐dependent *p*‐polarized PL measurement. As shown in **Figure** **4**, DPPM‐STAC, DPPM‐SXAC, and DPPM‐SFAC exhibit high Θ_//_ values of 80%, 82%, and 82%, indicating these molecules prefer horizontal dipole orientation. But DPPM‐DPAC and DPPM‐DMAC only have low Θ_//_s of 69% and 66%, respectively, close to those of purely isotropic chromophores. The low Θ_//_s are probably caused by the major *z* component of transition dipole moment vectors, as revealed by the simulation of transition dipole moment in S_1_ state (Figure 4). These results clearly demonstrate that the spiro‐structured acridine derivatives can significantly promote horizontal dipole orientation. On the other hand, the direction of transition dipole moment vector of DPPM‐SXAC is almost identical to that of DP‐SXAC, and the Θ_//_ of DPPM‐SXAC is also close to DP‐SXAC (79%) (Figure S11, Supporting Information), suggesting the introduction of benzoyl does not induce adverse effect on horizontal dipole orientation of these molecules.

**Figure 5:**
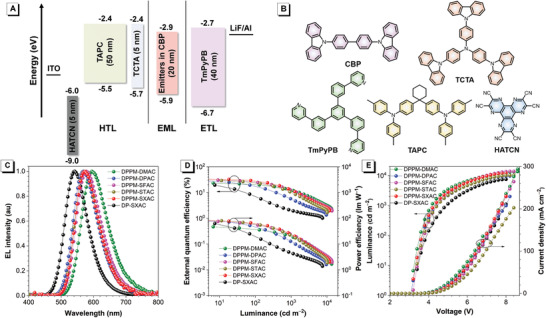
A) Device structure and energy level diagram. B) Molecular structures of the materials employed in the devices. C) EL spectra and plots of D) external quantum efficiency–luminance–power efficiency and E) luminance–voltage–current density of the devices.

The EL performances of DPPM‐based molecules are evaluated in multilayer OLEDs with a configuration of indium tin oxide (ITO)/2,3,6,7,10,11‐hexacyano‐1,4,5,8,9,12‐hexaazatriphenylene (HATCN, 5 nm)/1,10‐bis(di‐4‐tolylaminophenyl) cyclohexane (TAPC, 50 nm)/*tris*[4‐(carbazol‐9‐yl)phenyl]amine (TCTA, 5 nm)/emitting layers (EMLs, 20 nm)/1,3,5‐tri(*m*‐pyrid‐3‐yl‐phenyl)benzene (TmPyPB, 40 nm)/LiF (1 nm)/Al, where HATCN, TAPC, TCTA, TmPyPB, and LiF serve as hole injection, hole‐transporting, electron‐blocking, electron‐transporting, and electron injection layers, respectively (**Figure** **5**A,B). The doped films of DPPM‐based molecules in CBP host with varied doping concentrations function as EMLs, and CBP is chosen as the host because of its matched HOMO and LUMO energy levels. The key EL parameters are listed in **Table** **2**, and the dependence of EL performances on doping concentrations is summarized in Tables S3–S7 in the Supporting Information.

**Table 2:** EL performances of the OLEDs based on the new molecules

Emitter^a)^	*V*_on_ [V]	*L*_max_ [cd m^–2^]	*η*_C,max_ [cd A^–1^]	*η*_P,max_ [lm W^–1^]	*η*_ext,max_ [%]	*Φ*_out_ [%]	*λ*_EL_ [nm]	CIE (*x*, *y*)
DPPM‐DMAC	3.1	13 500	47.9	47.0	23.1	29	596	(0.56, 0.44)
DPPM‐DPAC	3.1	9610	85.0	83.4	27.3	31	568	(0.47, 0.52)
DPPM‐SFAC	3.1	13 810	82.1	77.0	30.6	37	578	(0.51, 0.49)
DPPM‐STAC	3.3	10 230	87.4	80.7	29.3	35	574	(0.48, 0.51)
DPPM‐SXAC	3.1	11 800	87.4	85.8	33.5	36	572	(0.49, 0.50)
DP‐SXAC	3.3	7480	68.1	62.9	19.5	35	542	(0.33, 0.58)

^a)^ Abbreviations: *V*_on_ = turn‐on voltage at 1 cd m^−2^; *L*_max_ = maximum luminance; *η*_C,max_ = maximum current efficiency; *η*_P,max_ = maximum power efficiency; *η*_ext,max_ = maximum external quantum efficiency; *Φ*_out_ = optical outcoupling efficiency; *λ*_EL_ = EL peak; CIE = Commission Internationale de I'Eclairage coordinates.

**Figure 6:**
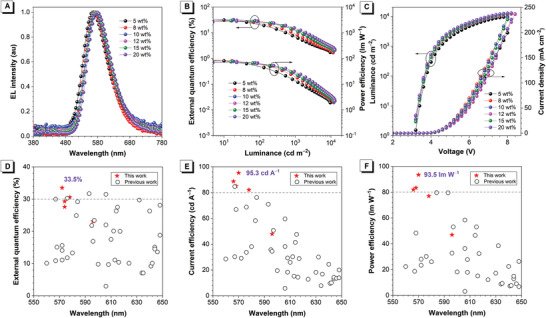
A) EL spectra and plots of B) external quantum efficiency–luminance–power efficiency and C) luminance–voltage–current density of doped OLEDs based on DPPM‐SXAC with varied doping concentrations. D) External quantum efficiency, E) current efficiency, and F) power efficiency versus EL peak wavelength of high‐efficiency orange to red TADF OLEDs reported in the literature.

The EL performances of the multilayer OLEDs employing DPPM‐based molecules with a doping concentration of 10 wt% are displayed in Figure 5C–E. These devices can be turned on at low voltages of 3.1 V and radiate orange–red EL emissions with peaks at 596, 568, 578, 574, and 572 nm for DPPM‐DMAC, DPPM‐DPAC, DPPM‐SFAC, DPPM‐STAC, and DPPM‐SXAC, respectively, corresponding to Commission Internationale de I'Eclairage coordinates of (0.56, 0.44), (0.47, 0.52), (0.51, 0.49), (0.48, 0.51), and (0.49, 0.50), respectively. But the device of DP‐SXAC exhibits green EL emission at 542 nm, which is blue‐shifted by 30 nm relative to that of DPPM‐SXAC. These devices show maximum luminance of 9610‒13 500 cd m^–2^ and provide excellent peak *η*_ext_ (*η*_ext,max_), current efficiency (*η*_C,max_), power efficiency (*η*_P,max_) of 23.1%, 47.9 cd A^−1^, and 47.0 lm W^−1^ for DPPM‐DMAC, 27.3%, 85.0 cd A^−1^, and 83.4 lm W^−1^ for DPPM‐DPAC, 30.6%, 82.1 cd A^−1^, and 77.0 lm W^−1^ for DPPM‐SFAC, 29.3%, 87.4 cd A^−1^, and 80.7 lm W^−1^ for DPPM‐STAC. And more impressively high EL efficiencies of 33.5%, 87.4 cd A^−1^, and 85.8 lm W^−1^ are achieved in DPPM‐SXAC device, which are significantly improved relative to those of DP‐SXAC device (19.5%, 68.1 cd A^−1^, and 62.9 lm W^−1^). Besides, DPPM‐SXAC can function efficiently in a concentration range of 5‒20 wt% (**Figure** **6**A–C and Table S6, Supporting Information). At a doping concertation of 8 wt%, its device provides *η*_ext,max_, *η*_C,max_, *η*_P,max_ of 31.8%, 95.3 cd A^−1^, and 93.5 lm W^−1^, respectively. To the best of our knowledge, the outstanding *η*_ext,max_ of 33.5%, *η*_C,max_ of 95.3 cd A^−1^, and *η*_P,max_ of 93.5 lm W^−1^ of DPPM‐SXAC device are the best efficiencies for orange–red TADF OLEDs reported so far (Figure 6D–F and Table S8, Supporting Information).

Based on film thickness, reflex index, and Θ_//_s,^[^ ^50^ ^]^ the *η*_out_s of DPPM‐STAC, DPPM‐SXAC, and DPPM‐SFAC are calculated to be 35%, 36%, and 37%, respectively, which are larger than those of DPPM‐DPAC (31%) and DPPM‐DMAC (29%). In addition, according to the formula of *η*_ext_ = *γ* × *Φ*_PL_ × *η*_eue_ × *η*_out_, in which *γ* is the charge balance factor (ideally *γ* = 1.0) and *η*_eue_ is the exciton utilization efficiency, the device of DPPM‐SXAC shows a highest *η*_eue_ of 98.9%, thanks to the fastest RISC.

**Figure 7:**
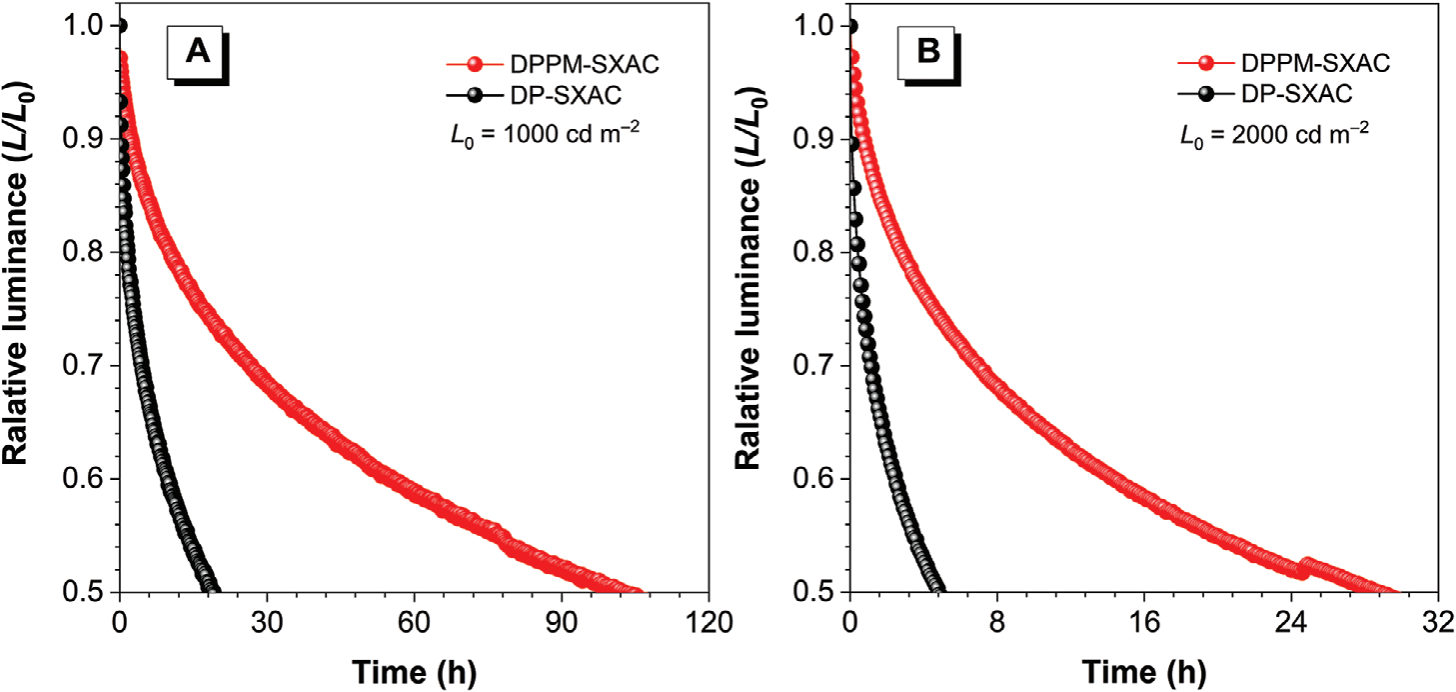
Device lifetimes of doped devices of DPPM‐SXAC and DP‐SXAC with initial luminance of A) 1000 cd m^‒2^ and B) 2000 cd m^‒2^. Device configuration: ITO/MoO_3_ (6 nm)/*m*CBP (40 nm)/10 wt% emitter: DIC‐TRZ (30 nm)/TPBi (50 nm)/LiF (1 nm)/Al.

On the other side, to evaluate the impact of benzoyl on the operational lifetime of the device, the device lifetimes of DPPM‐SXAC and DP‐SXAC are comparatively studied in identical device configurations of ITO/MoO_3_ (6 nm)/*m*CBP (40 nm)/10 wt% emitter: DIC‐TRZ (30 nm)/TPBi (50 nm)/LiF (1 nm)/Al, in which MoO_3_ is hole injection layer, *m*CBP (3,3’‐di(carbazol‐9‐yl)biphenyl) is hole‐transporting layer, DIC‐TRZ (2,4‐diphenyl‐6‐bis(12‐phenylindolo)[2,3‐a] carbazole‐11‐yl)‐1,3,5‐triazine) is the host of both emitters, and TPBi (1,3,5‐*tris*(*N*‐phenylbenzimidazol‐2‐yl)benzene) is electron‐transporting layer. The device of DPPM‐SXAC shows half‐life (LT50) values of 101 and 19 h at initial luminance of 1000 and 2000 cd m^‒2^, respectively, which are apparently longer than those of PM‐SXAC‐based device (29 and 5 h) (**Figure** **7**). To further evaluate the operational stability of both molecules, additional devices are fabricated with more stable functional layers by commercial company. The LT50 value of DPPM‐SXAC‐based device is ≈18 000 h at an initial luminance of 100 cd m^−2^, being about threefold higher than that of DP‐SXAC‐based device (≈6000 h) (Figure S12, Supporting Information). Although the device stability of these DPPM‐based molecules has not been fully optimized, these preliminary results suggest that the presence of benzoyl group will not bring about apparent negative effects but can improve device stability to some extent.

## 3 Conclusion

In summary, a series of orange–red delayed fluorescence molecules DPPM‐DMAC, DPPM‐DPAC, DPPM‐SFAC, DPPM‐STAC, and DPPM‐SXAC are successfully developed by fusing strong DPPM acceptor with acridine‐based donors. The introduction of benzoyl not only strengthens ICT effect but also enhances SOC matrix element and decreases Δ*E*_ST_ to promote RISC, leading to prominent delayed fluorescence with greatly red‐shifted emission peaks, enhanced *Φ*_PL_s, and shortened delayed lifetimes. On the other hand, the horizontal dipole orientation of the molecule can be facilitated by creating a spiro‐structure at the 9‐position of acridine, which is favored to improve Θ_//_s and *Φ*_out_s. And the presence of benzoyl induces no adverse effect on horizontal dipole orientation. This rational molecular engineering simultaneously endows DPPM‐SXAC with efficient red–orange delayed fluorescence, a high *Φ*_PL_ of 94%, and a large *Φ*_out_ of 36% in CBP host. As a result, DPPM‐SXAC realizes superb EL performance with *η*_ext,max_, *η*_C,max_, and *η*_P,max_ of up to 33.5%, 95.3 cd A^−1^, and 93.5 lm W^−1^, respectively. To the best of our knowledge, the outstanding EL efficiencies represent the best device performance among previously reported orange–red TADF OLEDs. Moreover, the preliminary results also suggest that the presence of benzoyl can improve device stability to some extent. It is envisioned that the proposed molecular design will bring about more efficient TADF materials for high‐performance OLEDs.

## Conflict of Interest

The authors declare no conflict of interest.

## Supporting information

Supporting Information

## Data Availability

The data that support the findings of this study are available from the corresponding author upon reasonable request.

## References

[1] C. W. Tang , S. A. VanSlyke , Appl. Phys. Lett. 1987, 51, 913.

[2] S. K. Jeon , H. L. Lee , K. S. Yook , J. Y. Lee , Adv. Mater. 2019, 31, 1803524. 10.1002/adma.201803524 30907464

[3] W. Zeng , H.‐Y. Lai , W.‐K. Lee , M. Jiao , Y.‐J. Shiu , C. Zhong , S. Gong , T. Zhou , G. Xie , M. Sarma , K.‐T. Wong , C.‐C. Wu , C. Yang , Adv. Mater. 2018, 30, 1704961. 10.1002/adma.201704961 29218854

[4] F.‐M. Xie , P. Wu , S.‐J. Zou , Y.‐Q. Li , T. Cheng , M. Xie , J.‐X. Tang , X. Zhao , Adv. Electron. Mater. 2020, 6, 1900843.

[5] Z. Yang , Z. Mao , Z. Xie , Y. Zhang , S. Liu , J. Zhao , J. Xu , Z. Chi , M. P. Aldred , Chem. Soc. Rev. 2017, 46, 915. 28117864 10.1039/c6cs00368k

[6] J. Guo , X.‐L. Li , H. Nie , W. Luo , S. Gan , S. Hu , R. Hu , A. Qin , Z. Zhao , S.‐J. Su , B. Z. Tang , Adv. Funct. Mater. 2017, 27, 1606458.

[7] J. Huang , H. Nie , J. Zeng , Z. Zhuang , S. Gan , Y. Cai , J. Guo , S.‐J. Su , Z. Zhao , B. Z. Tang , Angew. Chem., Int. Ed. 2017, 56, 12971. 10.1002/anie.201706752 28833917

[8] J.‐X. Chen , K. Wang , C.‐J. Zheng , M. Zhang , Y.‐Z. Shi , S.‐L. Tao , H. Lin , W. Liu , W.‐W. Tao , X.‐M. Ou , X.‐H. Zhang , Adv. Sci. 2018, 5, 1800436. 10.1002/advs.201800436 PMC6145404 30250791

[9] H. Liu , J. Zeng , J. Guo , H. Nie , Z. Zhao , B. Z. Tang , Angew. Chem., Int. Ed. 2018, 57, 9290. 10.1002/anie.201802060 29856500

[10] R. Furue , K. Matsuo , Y. Ashikari , H. Ooka , N. Amanokura , T. Yasuda , Adv. Opt. Mater. 2018, 6, 1701147.

[11] J. Li , T. Nakagawa , J. MacDonald , Q. Zhang , H. Nomura , H. Miyazaki , C. Adachi , Adv. Mater. 2013, 25, 3319. 23670919 10.1002/adma.201300575

[12] B. Wang , X. Qiao , Z. Yang , Y. Wang , S. Liu , D. Ma , Q. Wang , Org. Electron. 2018, 59, 32.

[13] H. Uoyama , K. Goushi , K. Shizu , H. Nomura , C. Adachi , Nature 2012, 492, 234. 23235877 10.1038/nature11687

[14] W. Li , M. Li , W. Li , Z. Xu , L. Gan , K. Liu , N. Zheng , C. Ning , D. Chen , Y.‐C. Wu , S.‐J. Su , ACS Appl. Mater. Interfaces 2021, 13, 5302. 33470809 10.1021/acsami.0c19302

[15] T.‐L. Wu , M.‐J. Huang , C.‐C. Lin , P.‐Y. Huang , T.‐Y. Chou , R.‐W. Chen‐Cheng , H.‐W. Lin , R.‐S. Liu , C.‐H. Cheng , Nat. Photonics 2018, 12, 235.

[16] Y.‐K. Chen , J. Jayakumar , C.‐M. Hsieh , T.‐L. Wu , C.‐C. Liao , J. Pandidurai , C.‐L. Ko , W.‐Y. Hung , C.‐H. Cheng , Adv. Mater. 2021, 33, 2008032. 10.1002/adma.202008032 34297444

[17] S. Wang , X. Yan , Z. Cheng , H. Zhang , Y. Liu , Y. Wang , Angew. Chem., Int. Ed. 2015, 54, 13068. 10.1002/anie.201506687 26480338

[18] Y.‐L. Zhang , Q. Ran , Q. Wang , Y. Liu , C. Hanisch , S. Reineke , J. Fan , L.‐S. Liao , Adv. Mater. 2019, 31, 1902368. 10.1002/adma.201902368 31490581

[19] T. Chen , C.‐H. Lu , Z. Chen , X. Gong , C.‐C. Wu , C. Yang , Chem. ‐ Eur. J. 2021, 27, 3151. 33241622 10.1002/chem.202004719

[20] J. H. Kim , J. H. Yun , J. Y. Lee , Adv. Opt. Mater. 2018, 6, 1800255.

[21] C. Li , R. Duan , B. Liang , G. Han , S. Wang , K. Ye , Y. Liu , Y. Yi , Y. Wang , Angew. Chem., Int. Ed. 2017, 56, 11525. 10.1002/anie.201706464 28718216

[22] J.‐X. Chen , Y.‐F. Xiao , K. Wang , D. Sun , X.‐C. Fan , X. Zhang , M. Zhang , Y.‐Z. Shi , J. Yu , F.‐X. Geng , C.‐S. Lee , X.‐H. Zhang , Angew. Chem., Int. Ed. 2021, 60, 2478. 10.1002/anie.202012070 33080106

[23] Z. Li , D. Yang , C. Han , B. Zhao , H. Wang , Y. Man , P. Ma , P. Chang , D. Ma , H. Xu , Angew. Chem., Int. Ed. 2021, 60, 14846. 10.1002/anie.202103070 33871909

[24] C. Chen , R. Huang , A. S. Batsanov , P. Pander , Y.‐T. Hsu , Z. Chi , F. B. Dias , M. R. Bryce , Angew. Chem., Int. Ed. 2018, 57, 16407. 10.1002/anie.201809945 30339314

[25] J. Xue , Q. Liang , R. Wang , J. Hou , W. Li , Q. Peng , Z. Shuai , J. Qiao , Adv. Mater. 2019, 31, 1808242. 10.1002/adma.201808242 31081199

[26] X. Gong , P. Li , Y.‐H. Huang , C.‐Y. Wang , C.‐H. Lu , W.‐K. Lee , C. Zhong , Z. Chen , W. Ning , C.‐C. Wu , S. Gong , C. Yang , Adv. Funct. Mater. 2020, 30, 1908839.

[27] X. Zeng , Y.‐H. Huang , S. Gong , P. Li , W.‐K. Lee , X. Xiao , Y. Zhang , C. Zhong , C.‐C. Wu , C. Yang , Mater. Horiz. 2021, 8, 2286. 34846432 10.1039/d1mh00613d

[28] C.‐M. Hsieh , T.‐L. Wu , J. Jayakumar , Y.‐C. Wang , C.‐L. Ko , W.‐Y. Hung , T.‐C. Lin , H.‐H. Wu , K.‐H. Lin , C.‐H. Lin , S. Hsieh , C.‐H. Cheng , ACS Appl. Mater. Interfaces 2020, 12, 23199. 32326694 10.1021/acsami.0c03711

[29] I. S. Park , S. Y. Lee , C. Adachi , T. Yasuda , Adv. Funct. Mater. 2016, 26, 1813.

[30] T. Chen , C.‐H. Lu , C.‐W. Huang , X. Zeng , J. Gao , Z. Chen , Y. Xiang , W. Zeng , Z. Huang , S. Gong , C.‐C. Wu , C. Yang , J. Mater. Chem. C 2019, 7, 9087.

[31] D.‐G. Chen , T.‐C. Lin , C.‐L. Chen , Y.‐T. Chen , Y.‐A. Chen , G.‐H. Lee , P.‐T. Chou , C.‐W. Liao , P.‐C. Chiu , C.‐H. Chang , Y.‐J. Lien , Y. Chi , ACS Appl. Mater. Interfaces 2018, 10, 12886. 29582654 10.1021/acsami.8b00053

[32] X. Cai , X. Li , G. Xie , Z. He , K. Gao , K. Liu , D. Chen , Y. Cao , S.‐J. Su , Chem. Sci. 2016, 7, 4264. 30155073 10.1039/c6sc00542j PMC6013828

[33] T. Yang , Z. Cheng , Z. Li , J. Liang , Y. Xu , C. Li , Y. Wang , Adv. Funct. Mater. 2020, 30, 2002681.

[34] D. Karthik , Y. H. Jung , H. Lee , S. Hwang , B.‐M. Seo , J.‐Y. Kim , C. W. Han , J. H. Kwon , Adv. Mater. 2021, 33, 2007724. 10.1002/adma.202007724 33792077

[35] Y.‐Y. Wang , K.‐N. Tong , K. Zhang , C.‐H. Lu , X. Chen , J.‐X. Liang , C.‐K. Wang , C.‐C. Wu , M.‐K. Fung , J. Fan , Mater. Horiz. 2021, 8, 1297. 34821922 10.1039/d1mh00028d

[36] S. Shao , L. Wang , Aggregate 2020, 1, 45.

[37] Z. Cai , X. Wu , H. Liu , J. Guo , D. Yang , D. Ma , Z. Zhao , B. Z. Tang , Angew. Chem., Int. Ed. 2021, 60, 23635. 10.1002/anie.202111172 34459540

[38] C. M. Marian , J. Phys. Chem. C 2016, 120, 3715.

[39] X. Cai , B. Gao , X.‐L. Li , Y. Cao , S.‐J. Su , Adv. Funct. Mater. 2016, 26, 8042.

[40] Q. Peng , Z. Shuai , Aggregate 2021, 2, e91.

[41] T.‐A. Lin , T. Chatterjee , W.‐L. Tsai , W.‐K. Lee , M.‐J. Wu , M. Jiao , K.‐C. Pan , C.‐L. Yi , C.‐L. Chung , K.‐T. Wong , C.‐C. Wu , Adv. Mater. 2016, 28, 6976. 27271917 10.1002/adma.201601675

[42] U. Balijapalli , Y.‐T. Lee , B. S. B. Karunathilaka , G. Tumen‐Ulzii , M. Auffray , Y. Tsuchiya , H. Nakanotani , C. Adachi , Angew. Chem., Int. Ed. 2021, 60, 19364. 10.1002/anie.202106570 34155775

[43] S. Zhang , L. Yao , Q. Peng , W. Li , Y. Pan , R. Xiao , Y. Gao , C. Gu , Z. Wang , P. Lu , F. Li , S. Su , B. Yang , Y. Ma , Adv. Funct. Mater. 2015, 25, 1755.

[44] H. Zhang , B. Zhang , Y. Zhang , Z. Xu , H. Wu , P.‐A. Yin , Z. Wang , Z. Zhao , D. Ma , B. Z. Tang , Adv. Funct. Mater. 2020, 30, 2002323.

[45] H. Noda , X.‐K. Chen , H. Nakanotani , T. Hosokai , M. Miyajima , N. Notsuka , Y. Kashima , J.‐L. Brédas , C. Adachi , Nat. Mater. 2019, 18, 1084. 31477903 10.1038/s41563-019-0465-6

[46] L.‐S. Cui , A. J. Gillett , S.‐F. Zhang , H. Ye , Y. Liu , X.‐K. Chen , Z.‐S. Lin , E. W. Evans , W. K. Myers , T. K. Ronson , H. Nakanotani , S. Reineke , J.‐L. Bredas , C. Adachi , R. H. Friend , Nat. Photonics 2020, 14, 636.

[47] Y. Hong , J. W. Y. Lam , B. Z. Tang , Chem. Soc. Rev. 2011, 40, 5361. 21799992 10.1039/c1cs15113d

[48] B. Chen , B. Liu , J. Zeng , H. Nie , Y. Xiong , J. Zou , H. Ning , Z. Wang , Z. Zhao , B. Z. Tang , Adv. Funct. Mater. 2018, 28, 1803369.

[49] C. Wang , L. Li , X. Zhan , Z. Ruan , Y. Xie , Q. Hu , S. Ye , Q. Li , Z. Li , Sci. Bull. 2016, 61, 1746.

[50] J.‐K. Hwang , H.‐Y. Ryu , Y.‐H. Lee , Phys. Rev. B 1999, 60, 4688.

