# Supplementary material for: High‐Performance Orange–Red Organic Light‐Emitting Diodes with External Quantum Efficiencies Reaching 33.5% based on Carbonyl‐Containing Delayed Fluorescence Molecules
Source: Adv Sci (Weinh). 2021 Dec 19;9(3):2104435. doi: 10.1002/advs.202104435 (PMC8787409; doi:10.1002/advs.202104435)
Supplement: Supplementary file 1 — Supporting Information [file ADVS-9-2104435-s001.pdf]

ADVANCED SCIENCE
Open Access

Supporting Information

for *Adv. Sci.*, DOI: 10.1002/advs.202104435

High-Performance Orange-Red Organic Light-Emitting Diodes with External Quantum Efficiencies Reaching 33.5% Based on Carbonyl-Containing Delayed Fluorescence Molecules

Ruming Jiang, Xing Wu, Hao Liu, Jingjing Guo, Dijia Zou, Zujin Zhao, and Ben Zhong Tang*

Supporting Information

General information

All the chemicals and reagents were purchased from commercial sources and used as received without further purification. ^{1}H and ^{13}C NMR spectra were measured on a Bruker AV 500 spectrometer in $C_2D_2Cl_4$ or CD_2Cl_2 at room temperature. High-resolution mass spectra (HRMS) were recorded on an Agilent1290/Bruker maXis impact mass spectrometer. UV-vis absorption spectra were measured on a Shimadzu UV-2600 spectrophotometer. Photoluminescence (PL) spectra were recorded on a Horiba Fluoromax-4 spectrofluorometer. PL quantum yields were measured using a Hamamatsu absolute PL quantum yield spectrometer C11347 Quantaurus_QY. Temperature-dependent transient PL decay in films were measured in Edinburgh Instruments FLS980 spectrometer. The prompt fluorescence lifetimes were measured by using time-correlated single photon counting (TCSPC) mode with a picosecond light source and delayed fluorescence lifetimes were measured by using multi-channel scaling (MCS) mode with a variable pulse lasers (VPL) light source. Transient PL decay spectra of materials in solutions were measured using Quantaurus-Tau fluorescence lifetime measurement system (C11367-03, Hamamatsu Photonics Co., Japan). Thermal gravimetric analysis (TGA) data were collected from a TG209F1 under nitrogen protection at a heating rate of 20 K min^{-1}. Differential scanning calorimetric (DSC) analysis were performed on a DSC 214 Polymer under dry nitrogen at a heating rate of 10 °C min^{-1}. The ground-state geometries were optimized using the density function theory (DFT) method with PBE0 functional at the basis set level of 6-31G**, and the ΔE_{ST} values were calculated by time-dependent DFT (TD-DFT) method at the PBE0/6-31G** level.[1] The SOC matrix elements between singlet and triplet states were calculated with the PySOC program. All the

calculations were performed using Gaussian16 package. Cyclic voltammogram was measured in a solution of tetra-*n*-butylammonium hexafluorophosphate (0.1 M) in dichloromethane (oxidation) and *N, N*-dimethylformamide (reduction) containing the sample at a scan rate of 50 mV s^{-1}. Three-electrode system (Ag/Ag^{+}, platinum wire and glassy carbon electrode as reference, counter and work electrode respectively) was used in the CV method. HOMO = –[E_{ox} + 4.8] eV, and LUMO = –[E_{re} + 4.8] eV. E_{ox} and E_{re} represent the onset oxidation and reduction potentials relative to ferrocene, respectively. For the measurement of orientation of emitting dipoles in molecules, a setup RSQX-01 made by the Changchun Ruoshui Technology Development Co., Ltd. was used. The dipole orientation of doped films was determined by angle-resolved and polarization resolved PL on a half quartz cylinder prism. A continuous-wave He:Cd laser (325 nm) with a fixed angle of 45° to the substrate was employed as excitation source. *p*-Polarized emitted light was detected at the respective peak wavelength of the PL spectrum of each film. The device operational lifetime was evaluated by commercial company.

OLED fabrication and characterization

The glass substrates precoated with a 90-nm layer of indium tin oxide (ITO) with a sheet resistance of 15~20 Ω per square were successively cleaned in ultrasonic bath of acetone, isopropanol, detergent and deionized water, respectively, taking 10 minutes for each step. Then, the substrates were totally dried in a 70 ^{o}C oven. Before the fabrication processes, in order to improve the hole injection ability of ITO, the substrates were treated by O_2 plasma for 10 minutes. The vacuum-deposited OLEDs were fabricated under a pressure of < 5×10^{-4} Pa in the Fangsheng OMV-FS450 vacuum deposition system. Organic materials, LiF and Al were deposited at rates of 1~2 A s^{-1}, 0.1 A s^{-1} and 5 A s^{-1}, respectively. The effective emitting area of the devices is 9 mm 2, determined by the overlap between anode and cathode. The luminance–voltage–current density characteristics and EL spectra were obtained via a Konica Minolta CS-200 Color and Luminance Meter and an Ocean Optics USB 2000+

spectrometer, along with a Keithley 2400 Source Meter. The external quantum efficiencies were estimated utilizing the normalized EL spectra and the current efficiencies of the devices, assuming that the devices were Lambertian emitters. All the characterizations were conducted at room temperature in ambient conditions without any encapsulation, as soon as the devices were fabricated.

Synthesis and characterization

The intermediates DPPM-Br and DP-Br were synthesized according to the methods in the literature.[2,22]

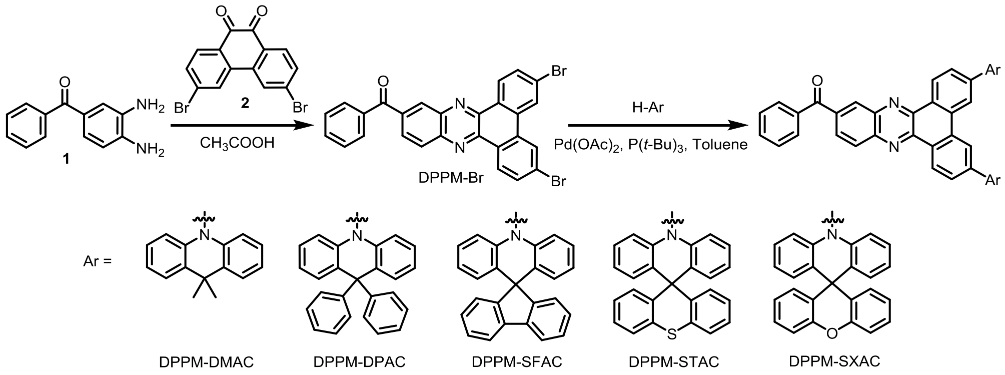

Scheme S1. Synthetic routes of DPPM derivatives.

(3,6-Bis(9,9-dimethylacridin-10(9*H*)-yl)dibenzo[*a*,*c*]phenazin-11-yl)(phenyl)methanone (DPPM-DMAC): A mixture of compound DPPM-Br (1.08 g, 2 mmol), 9,9-dimethyl-9,10-dihydroacridine (0.92 g, 4.4 mmol), $Pd(OAc)_2$ (13.4 mg, 0.06 mmol), P(*t*-Bu)$_3$ (48.5 mg, 0.24 mmol), sodium *tert*-butoxide (0.58 g, 6 mmol) and anhydrous toluene (80 mL) was stirred at 120 ^{o}C under nitrogen atmosphere for 24 h. After the reaction was completed, the mixture was cooled down to room temperature. The mixture was poured into 50 mL water, and extracted with CH_2Cl_2 (3 × 100 mL). The combined organic layers were dried over anhydrous $MgSO_4$. After filtration, the solvent was removed and the resultant crude product was purified by silica gel column chromatography with petroleum ether/dichloromethane (v/v = 2/1) as the eluent to afford compound DPPM-DMAC as an orange solid (1.10 g, yield 69%). ^{1}H NMR (500 MHz, CD_2Cl_2) δ (ppm) 9.77 (d, J = 8.5 Hz, 1H), 9.69 (d, J = 8.5 Hz, 1H), 8.77 (d, J = 1.7 Hz, 1H),

8.54 (d, J = 8.8 Hz, 1H), 8.48–8.47 (m, 2H), 8.40 (dd, J = 8.8, 1.9 Hz, 1H), 8.01–7.99 (m, 2H), 7.81–7.71 (m, 3H), 7.64–7.61 (m, 2H), 7.48–7.45 (m, 4H), 6.94–6.89 (m, 8H), 6.37–6.33 (m, 4H), 1.69 (d, J = 3.5 Hz, 12H). ^{13}C NMR (125 MHz, CD_2Cl_2) δ 196.89, 145.53, 145.30, 145.25, 144.68, 144.35, 142.78, 141.96, 139.92, 138.60, 135.96, 135.66, 134.23, 134.06, 133.07, 130.00, 131.59, 131.53, 131.46, 131.28, 131.25, 131.15, 131.14, 131.12, 130.98, 130.65, 129.89, 127.68, 127.54, 127.49, 126.76, 122.15, 122.11 115.47, 115.42, 37.28, 32.60. HRMS (ESI, *m/z*) calcd. for $C_{57}H_{42}N_4NaO$ [M + Na]$^+$: 821.3251; found: 821.3254.

(3,6-Bis(9,9-diphenylacridin-10(9*H*)-yl)dibenzo[*a,c*]phenazin-11-yl)(phenyl)methanone (DPPM-DPAC): The procedure was analogous to that described for DPPM-DMAC. Orange solid of DPPM-DPAC was obtained in 65% yield. ^{1}H NMR (500 MHz, CD_2Cl_2) δ (ppm) 9.63 (d, J = 8.3 Hz, 1H), 9.54 (d, J = 8.4 Hz, 1H), 8.73 (d, J = 1.6 Hz, 1H), 8.51 (d, J = 8.8 Hz, 1H), 8.37 (dd, J = 8.8, 1.9 Hz, 1H), 7.99–7.97 (m, 2H), 7.74–7.54 (m, 7H), 7.19–6.92 (m, 32H), 6.56–6.52 (m, 4H). ^{13}C NMR (125 MHz, CD_2Cl_2) δ 195.56, 146.39, 143.93, 143.86, 143.64, 143.32, 142.95, 142.16, 141.42, 138.56, 137.28, 134.07, 133.78, 132.89, 132.71, 131.61, 130.35, 130.24, 130.13, 130.00, 129.92, 129.82, 129.09, 128.77, 128.57, 127.65, 126.98, 126.50, 126.17, 120.57, 114.59, 56.84. HRMS (ESI, *m/z*) calcd. for $C_{77}H_{50}N_4NaO$ [M + Na]$^+$: 1069.3877; found: 1069.3883.

(3,6-Bi(10*H*-spiro[acridine-9,9'-fluoren]-10-yl)dibenzo[*a,c*]phenazin-11-yl)(phenyl)methanone (DPPM-SFAC): The procedure was analogous to that described for DPPM-DMAC. Orange solid of DPPM-SFAC was obtained in 75% yield. ^{1}H NMR (500 MHz, $C_2D_2Cl_4$) δ (ppm) 9.95 (d, J = 8.2 Hz, 1H), 9.88 (d, J = 8.2 Hz, 1H), 8.90 (d, J = 1.6 Hz, 1H), 8.77 (d, J = 4.2 Hz, 2H), 8.67 (d, J = 8.8 Hz, 1H), 8.50 (d, J = 8.7 Hz, 1H), 8.10–8.01 (m, 4H), 7.90–7.70 (m, 7H), 7.58–7.30 (m, 12H), 7.02–6.98 (m, 4H), 6.68–6.49 (m, 12H). ^{13}C NMR (125 MHz, $C_2D_2Cl_2$) δ 196.18, 156.64, 144.14, 144.06, 143.83, 143.53, 143.15, 141.56, 141.17, 139.33, 138.59, 137.18, 134.61, 134.32, 133.40, 133.12, 131.84, 130.43, 130.35, 130.32, 130.20, 128.93, 128.56, 128.12, 127.84, 127.58, 126.49, 125.98, 125.08, 125.05,

121.10, 120.22, 114.96, 56.87. HRMS (ESI, *m/z*) calcd. for $C_{77}H_{46}N_4NaO$ $[M + Na]^+$: 1065.3564; found: 1065.3562.

(3,6-Bi(10H-spiro[acridine-9,9'-thioxanthen]-10-yl)dibenzo[a,c]phenazin-11-yl)(phenyl)methanone (DPPM-STAC): The procedure was analogous to that described for DPPM-DMAC. Orange-red solid of DPPM-SXAC was obtained in 81% yield. ^{1}H NMR (500 MHz, CD_2Cl_4) δ (ppm) 9.94 (d, *J* = 8.4 Hz, 1H), 9.86 (d, *J* = 8.4 Hz, 1H), 8.89 (d, *J* = 1.7 Hz, 1H), 8.68–8.66 (m, 3H), 8.49 (dd, *J* = 8.8, 1.9 Hz, 1H), 8.09–8.08 (m, 2H), 7.99–7.93 (m, 2H), 7.83–7.80 (m, 1H), 7.72–7.69 (m, 2H), 7.35–6.74 (m, 28H), 6.49–6.46 (m, 4H). ^{13}C NMR (125 MHz, CD_2Cl_4) δ 196.16, 144.10, 143.88, 143.65, 143.45, 143.08, 142.43, 141.56, 138.64, 138.26, 137.16, 134.58, 134.29, 134.16, 133.41, 133.06, 132.53, 131.89, 131.15, 130.43, 128.93, 127.35, 127.27, 126.45, 124.88, 121.41, 114.5, 49.93. HRMS (ESI, *m/z*) calcd. for $C_{77}H_{47}N_4OS_2$ $[M + H]^+$: 1107.3186; found: 1107.3196.

(3,6-Bi(10*H*-spiro[acridine-9,9'-xanthen]-10-yl)dibenzo[*a,c*]phenazin-11-yl)(phenyl)methanone (DPPM-SXAC): The procedure was analogous to that described for DPPM-DMAC. Orange-red solid of DPPM-SXAC was obtained in 78% yield. ^{1}H NMR (500 MHz, CD_2Cl_2) δ (ppm) 9.88 (d, *J* = 8.4 Hz, 1H), 9.80 (d, *J* = 8.4 Hz, 1H), 8.81 (d, *J* = 1.6 Hz, 1H), 8.70–8.69 (m, 2H), 8.59 (d, *J* = 8.8 Hz, 1H), 8.44 (dd, *J* = 8.8, 1.9 Hz, 1H), 8.04–8.01 (m, 2H), 7.96–7.90 (m, 2H), 7.77–7.73 (m, 1H), 7.66–7.62 (m, 2H), 7.25–6.68 (m, 28H), 6.46–6.42 (m, 4H). ^{13}C NMR (125 MHz, CD_2Cl_2) δ 196.25, 149.09, 144.77, 144.45, 144.24, 144.02, 143.66, 142.29, 139.65, 139.44, 137.96, 135.30, 135.01, 133.65, 133.47, 133.03, 132.61, 132.31, 132.15, 131.02, 130.99, 130.92, 130.85, 130.79, 130.72, 130.61, 130.50, 129.29, 128.45, 127.78, 127.32, 124.29, 121.74, 116.69, 115.06, 45.36. HRMS (ESI, *m/z*) calcd. for $C_{77}H_{46}N_4NaO_3$ $[M + Na]^+$: 1097.3462; found: 1097.3471.

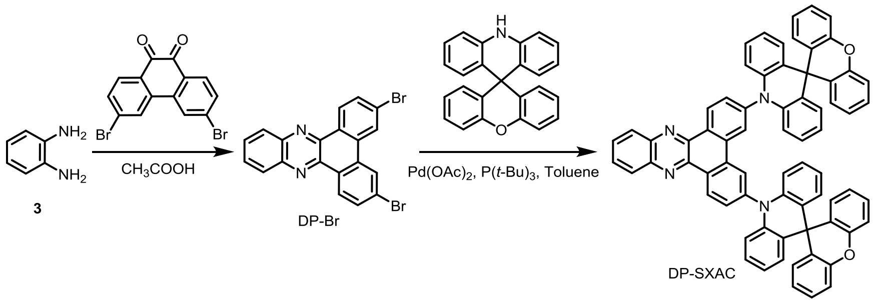

Scheme 2. Synthesis of DP-SXAC.

3,6-Bi(10*H*-spiro[acridine-9,9'-xanthen]-10-yl)dibenzo[*a*,*c*]phenazine (DP-SXAC): The procedure was analogous to that described for DPPM-DMAC. Green solid of DP-SXAC was obtained in 70% yield. ^{1}H NMR (500 MHz, CD_2Cl_2) δ (ppm) 9.87 (d, J = 8.4 Hz, 2H), 8.69 (s, 2H), 8.51–8.49 (m, 2H), 8.03–8.01 (m, 2H), 7.92 (d, J = 8.1 Hz, 2H), 7.24–6.68 (m, 28H), 6.44 (d, J = 8.2 Hz, 4H). ^{13}C NMR (125 MHz, CD_2Cl_2) δ 149.05, 143.87, 143.15, 142.53, 139.67, 134.84, 132.97, 132.59, 132.14, 132.08, 131.36, 131.17, 130.54, 130.45, 130.14, 128.39, 127.73, 127.17, 124.26, 121.66, 116.63, 115.05, 45.33. HRMS (ESI, *m/z*) calcd. for $C_{70}H_{42}N_4NaO_2$ $[M + Na]^+$: 993.3200; found: 993.3217.

Estimation of photophysical parameters

The quantum efficiencies and rate constants were determined using the following equations according to the following equations [a)]:

$\Phi_p = \Phi_F R_p$ (1)

$\Phi_d = \Phi_F R_d$ (2)

$k_F = \Phi_p/\tau_p$ (3)

$\Phi_F = k_F/(k_F + k_{IC})$ (4)

$\Phi_p = k_F/(k_F + k_{IC} + k_{ISC})$ (5)

$\Phi_{IC} = k_{IC}/(k_F + k_{IC} + k_{ISC})$ (6)

$\Phi_{ISC} = k_{ISC}/(k_F + k_{IC} + k_{ISC}) = 1 - \Phi_p - \Phi_{IC}$ (7)

$\Phi_{RISC} = \Phi_d/\Phi_{ISC}$ (8)

$k_{RISC} = (k_p k_d \Phi_d)/(k_{ISC}\Phi_p)$ (9)

$k_p = 1/\tau_p$; $k_d = 1/\tau_d$ (10)

[a] Abbreviations: Φ_{PL} = absolute photoluminescence quantum yield; τ_p and τ_d = lifetimes calculated from the prompt and delayed fluorescence decay, respectively; R_p and R_d = ratio of prompt component and delayed component and $R_d = 1 - R_p$; Φ_p and Φ_d = quantum yields from prompt and delayed components, respectively, determined from the total Φ_{PL} and the proportion of the integrated area of each component in the transient spectra to the total integrated area; Φ_{ISC} = intersystem crossing quantum yield; k_F = fluorescence decay rate; k_{IC} = internal conversion rate from S_1 to S_0 states; k_{ISC} = intersystem crossing rate from S_1 to T_1 states; k_{RISC} = rate constant of reverse intersystem crossing.

Additional data

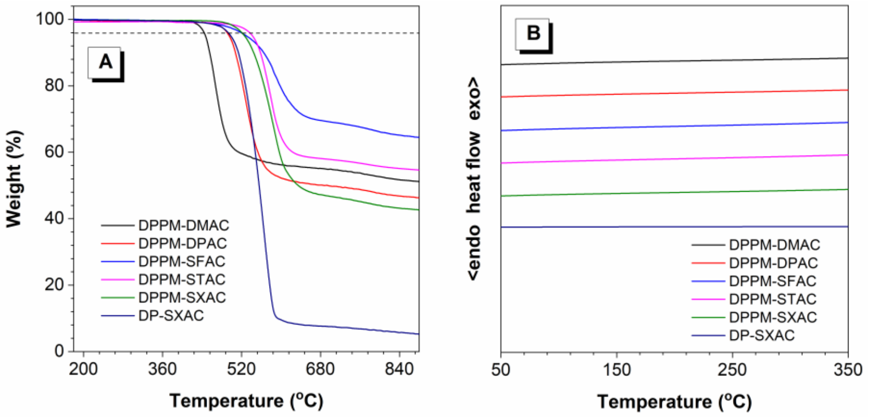

Figure S1. (A) TGA and (B) DSC thermograms of DPPM-DMAC, DPPM-DPAC, DPPM-SFAC, DPPM-STAC, DPPM-SXAC and DP-SXAC.

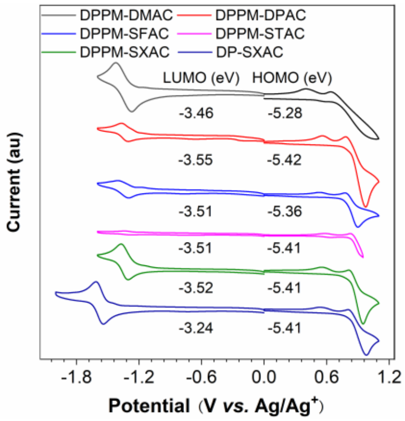

Figure S2. Cyclic voltammograms of DPPM-DMAC, DPPM-DPAC, DPPM-SFAC, DPPM-STAC, DPPM-SXAC and DP-SXAC.

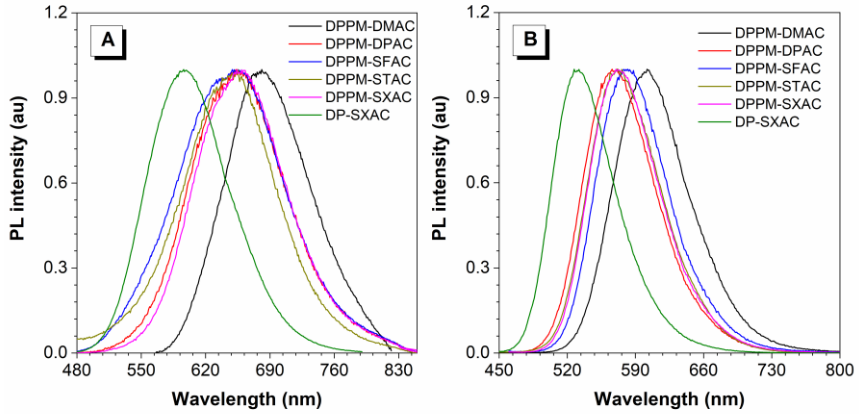

Figure S3. PL spectra of DPPM-DMAC, DPPM-DPAC, DPPM-SFAC, DPPM-STAC, DPPM-SXAC, DP-SXAC in (A) THF and (B) toluene solutions (10^{-5} M).

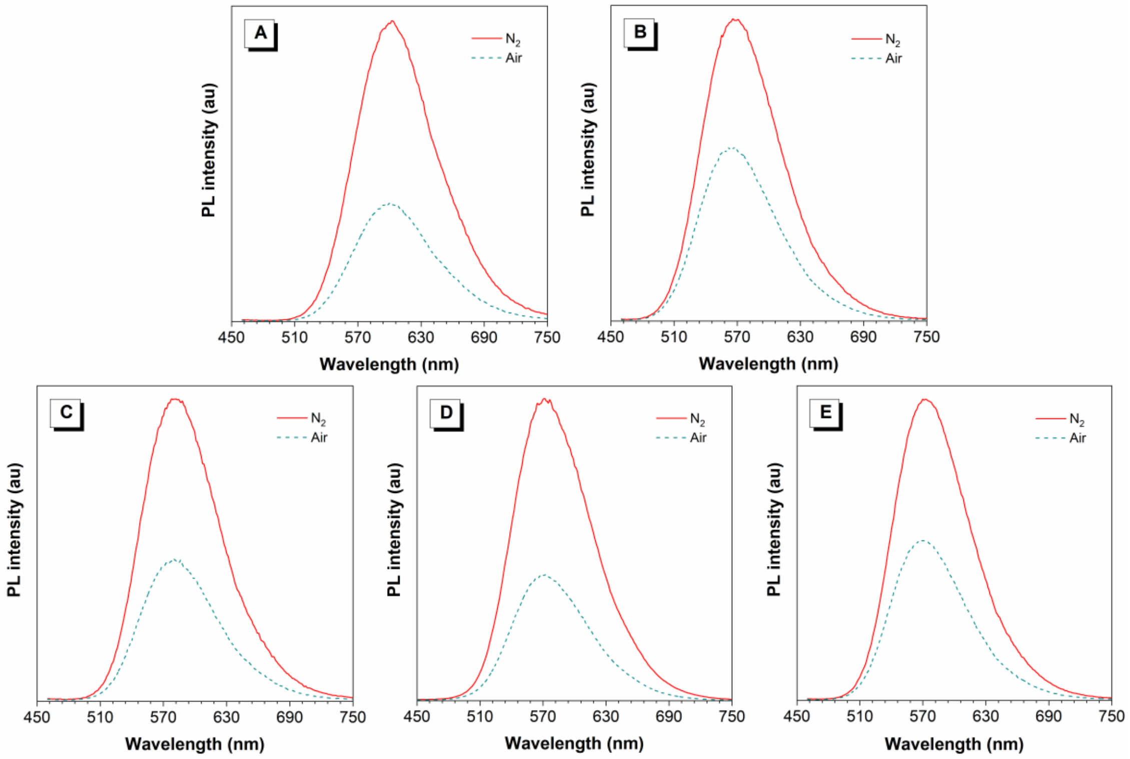

Figure S4. PL spectra of (A) DPPM-DMAC, (B) DPPM-DPAC, (C) DPPM-SFAC, (D) DPPM-STAC and (E) DPPM-SXAC in toluene solutions (10^{-5} M) at room temperature in N_2 and air.

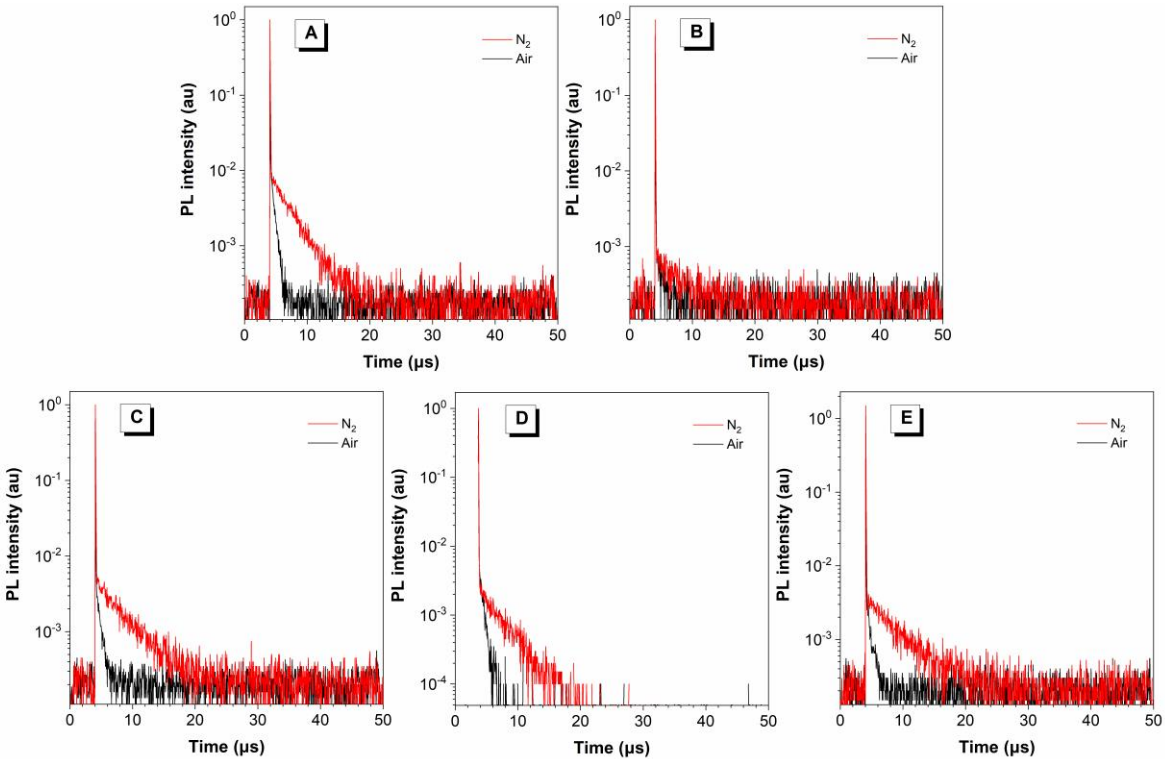

Figure S5. Transient PL decay spectra of (A) DPPM-DMAC, (B) DPPM-DPAC, (C) DPPM-SFAC, (D) DPPM-STAC and (E) DPPM-SXAC in toluene solutions (10^{-5} M) at room temperature in N_2 and air.

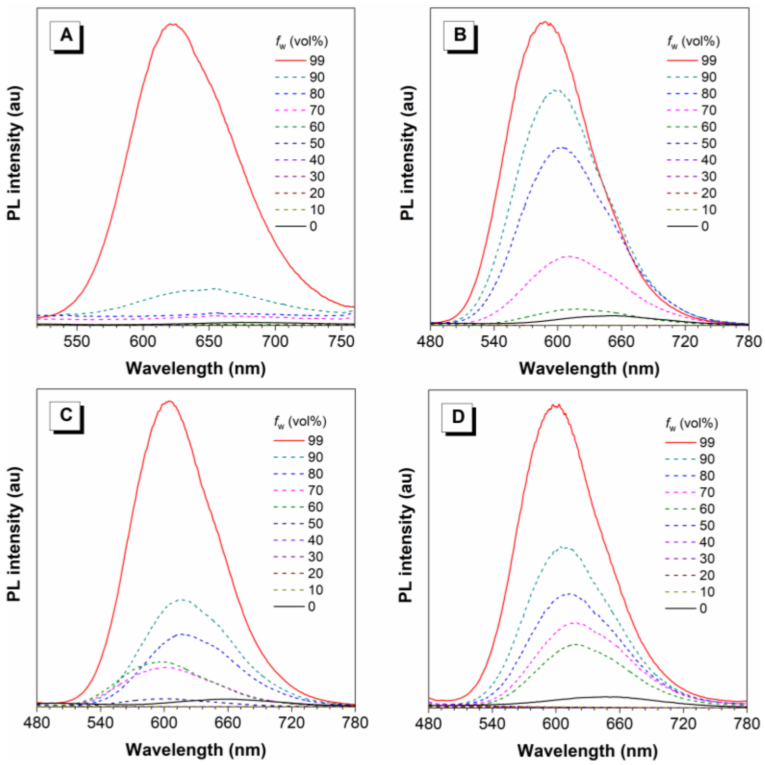

Figure S6. PL spectra of (A) DPPM-DMAC, (B) DPPM-DPAC, (C) DPPM-SFAC and (D) DPPM-STAC in THF/water mixtures with different water fractions (f_ws).

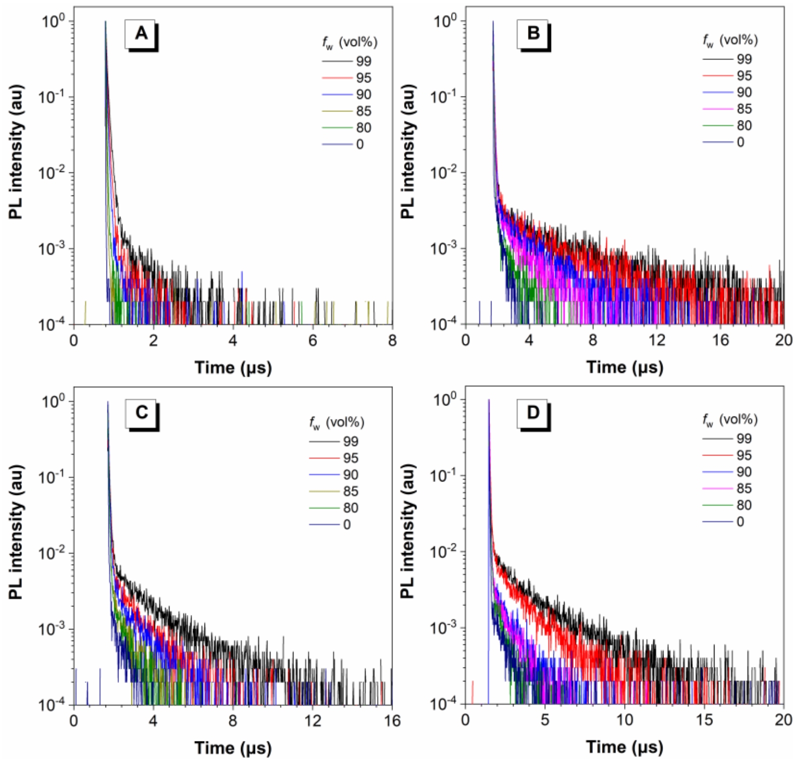

Figure S7. Transient PL decay spectra of (A) DPPM-DMAC, (B) DPPM-DPAC, (C) DPPM-SFAC and (C) DPPM-STAC in THF/water mixtures with different f_ws.

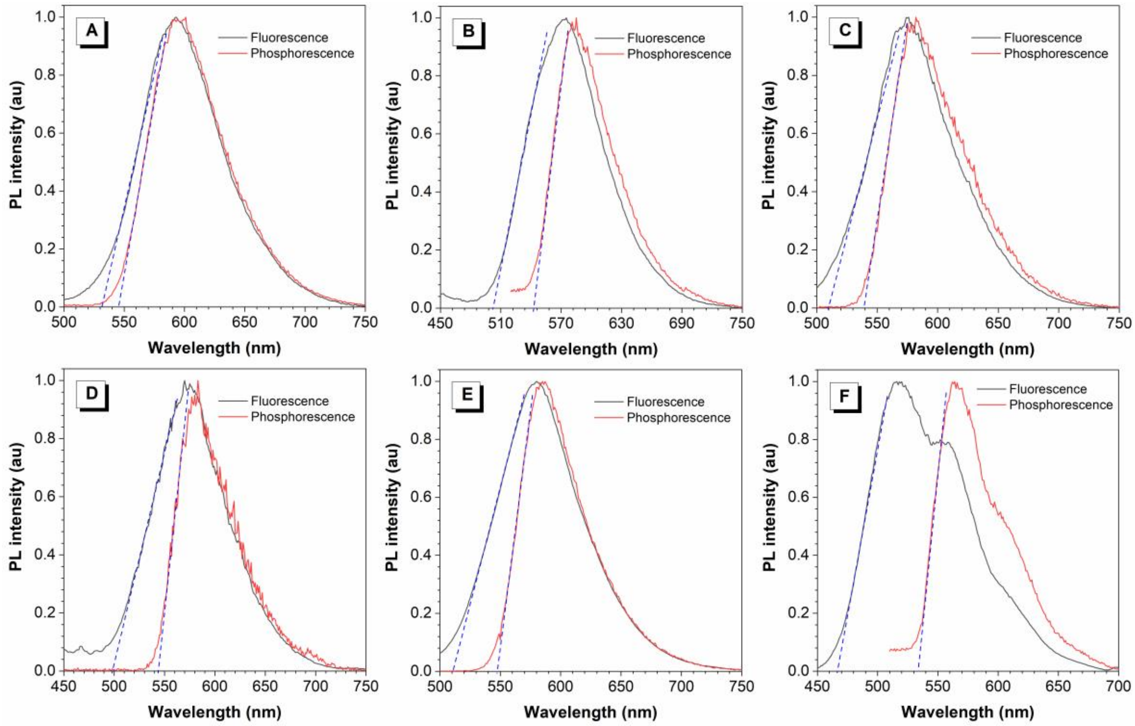

Figure S8. Fluorescence and phosphorescence spectra of (A) DPPM-DMAC, (B) DPPM-DPAC, (C) DPPM-SFAC, (D) DPPM-STAC, (E) DPPM-SXAC and (F) DP-SXAC doped in CBP films with a concentration of 10 wt% measured at 77 K.

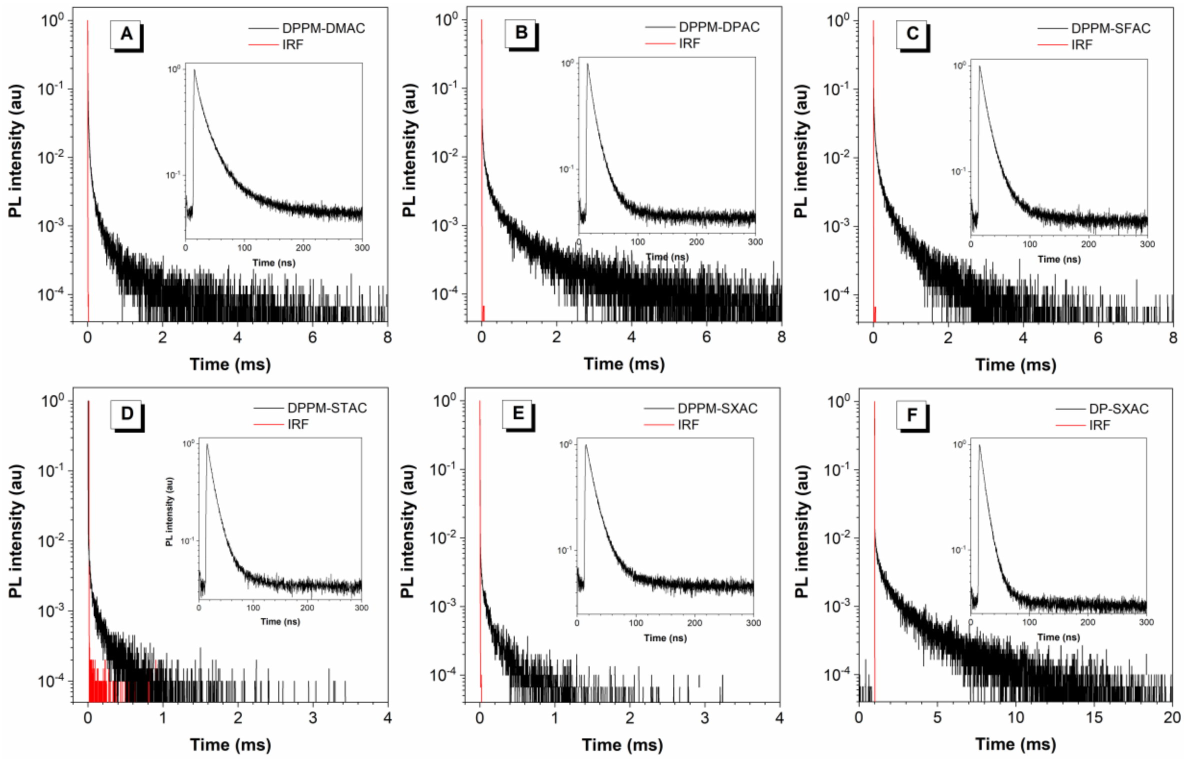

Figure S9. Transient PL decay spectra of (A) DPPM-DMAC, (B) DPPM-DPAC, (C) DPPM-SFAC, (D) DPPM-STAC, (E) DPPM-SXAC, and (F) DP-SXAC doped in CBP films with a concentration of 10 wt% measured at 300 K.

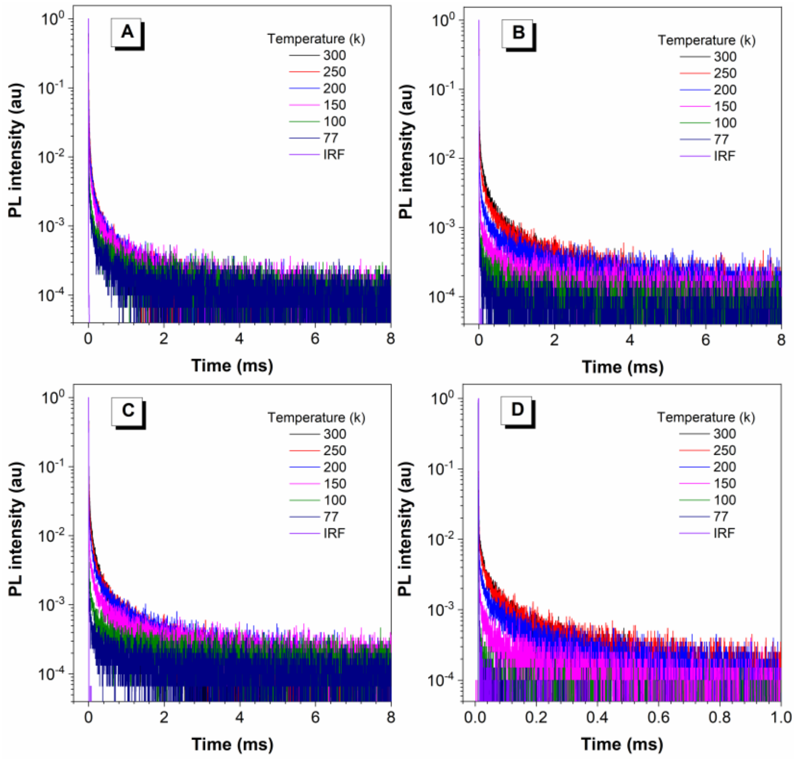

Figure S10. Temperature-dependent transient PL decay spectra of (A) DPPM-DMAC, (B) DPPM-DPAC, (C) DPPM-SFAC and (C) DPPM-STAC doped in CBP films with a concentration of 10 wt%.

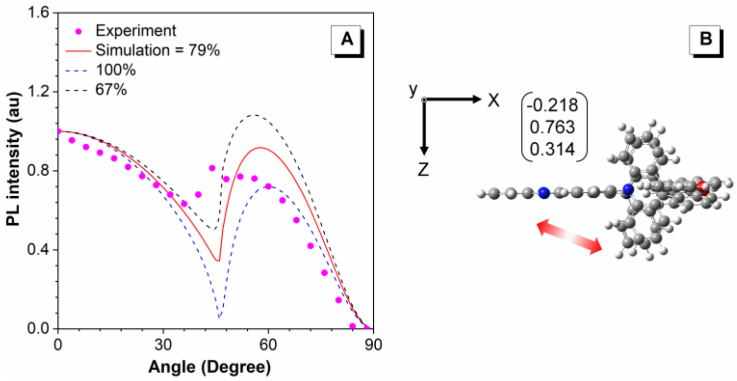

Figure S11. (A) Measured (symbols) *p*-polarized PL intensity (at PL peak wavelength) of the doped film of DP-SXAC in CBP host (10 wt%). (B) Simulated dipole moment vector for DP-SXAC.

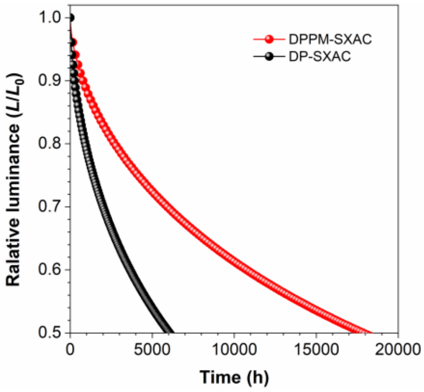

Figure S12. Device lifetimes of 10 wt% doped DPPM-SXAC and DP-SXAC devices with initial luminance of 100 cd m^{-2}. Device configuration: ITO/HT21: H09 (100 nm, 5 wt%)/HT21 (1800 nm)/HT18 (100 nm)/PH315: RD314 (200 nm, 2 wt%)/ET14 (400 nm)/ET20 (100 nm)/ET20: IN05 (100, 1 wt%)/Al.

Table S1. Photophysical parameters of these new molecules in solutions.

compound	λ_{abs} [nm]		λ_{PL} [nm]		Φ_{PL} [%]	
	THF	Toluene	THF	Toluene	THF	Toluene
DPPM-DMAC	402, 456	403, 461	682	603	1	44
DPPM-DPAC	400, 442	402, 445	656	568	3	34
DPPM-SFAC	401, 446	402, 460	656	582	1	29
DPPM-STAC	401, 445	401, 459	655	571	4	43
DPPM-SXAC	401, 445	401, 457	658	573	4	39
DP-SXAC	390, 427	390, 431	598	532	20	35

Table S2. Temperature-dependent photophysical parameters of doped films in CBP host with a concentration of 10 wt%.[a)]

		DPPM-DMAC	DPPM-DPAC	DPPM-SFAC	DPPM-STAC	DPPM-SXAC
τ_d [µs]	300 K	125	455	233	72	37
	250 K	149	643	419	73	29
	200 K	241	802	601	53	14
	150 K	320	762	762	34	4
	100 K	369	450	797	39	21
	77 K	309	1106	603	60	12
R_d [%]	300 K	41	35	46	30	19
	250 K	39	28	39	26	13
	200 K	33	14	30	13	8
	150 K	23	5	16	8	6
	100 K	10	2	6	6	1
	77 K	6	1	2	4	1

[a)] τ_d = lifetimes calculated from delayed fluorescence decay; R_d = ratio of delayed component.

Table S3. EL performances of devices based on DPPM-DMAC with different doping concentrations.

Concentration [wt%]	λ_{EL} [nm]	V_{on} [V]	L_{max} [cd m^{-2}]	$\eta_{C,max}$ [cd A^{-1}]	$\eta_{P,max}$ [lm W^{-1}]	$\eta_{ext,max}$ [%]	CIE (x, y)
10	596	3.1	13500	47.9	47.0	23.1	(0.56, 0.44)
15	600	2.9	15120	38.6	37.9	20.3	(0.58, 0.42)
20	610	2.9	15440	31.3	32.8	18.2	(0.59, 0.41)

Table S4. EL performances of devices based on DPPM-DPAC with different doping concentrations.

Concentration [wt%]	λ_{EL} [nm]	V_{on} [V]	L_{max} [cd m^{-2}]	$\eta_{C,max}$ [cd A^{-1}]	$\eta_{P,max}$ [lm W^{-1}]	$\eta_{ext,max}$ [%]	CIE (x, y)
10	568	3.1	9610	85.0	83.4	27.3	(0.47, 0.52)
15	574	3.1	10690	82.5	81.0	27.6	(0.49, 0.51)
20	578	2.9	11770	75.3	78.8	26.3	(0.50, 0.50)

Table S5. EL performances of devices based on DPPM-SFAC with different doping concentrations.

Concentration [wt%]	λ_{EL} [nm]	V_{on} [V]	L_{max} [cd m^{-2}]	$\eta_{C,max}$ [cd A^{-1}]	$\eta_{P,max}$ [lm W^{-1}]	$\eta_{ext,max}$ [%]	CIE (x, y)
5	570	3.5	10390	79.1	69.1	29.0	(0.48, 0.51)
10	578	3.1	13810	82.1	77.0	30.6	(0.51, 0.49)
15	584	3.1	16340	73.2	68.2	28.7	(0.53, 0.47)
20	588	3.1	17160	62.8	57.6	25.9	(0.54, 0.46)

Table S6. EL performances of devices based on DPPM-STAC with different doping concentrations.

Concentration [wt%]	λ_{EL} [nm]	V_{on} [V]	L_{max} [cd m^{-2}]	$\eta_{C,max}$ [cd A^{-1}]	$\eta_{P,max}$ [lm W^{-1}]	$\eta_{ext,max}$ [%]	CIE (x, y)
5	566	3.3	7640	88.7	82.0	27.9	(0.46, 0.53)
10	574	3.3	10230	87.4	80.7	29.3	(0.48, 0.51)
15	582	3.1	12290	69.5	64.2	25.5	(0.52, 0.48)
20	586	2.9	14550	61.5	58.3	23.9	(0.53, 0.47)

Table S7. EL performances of devices based on DPPM-SXAC with different doping concentrations.

Concentration [wt%]	λ_{EL} [nm]	V_{on} [V]	L_{max} [cd m^{-2}]	$\eta_{C,max}$ [cd A^{-1}]	$\eta_{P,max}$ [lm W^{-1}]	$\eta_{ext,max}$ [%]	CIE (x, y)
5	568	3.1	9610	86.7	82.1	31.0	(0.47, 0.52)
8	570	3.1	11480	95.3	93.5	31.8	(0.48, 0.52)
10	572	3.1	11800	87.4	85.8	33.5	(0.49, 0.50)
12	574	3.1	12060	84.8	83.3	31.6	(0.50, 0.50)
15	574	3.1	12050	80.1	74.0	29.3	(0.50, 0.50)
20	578	3.1	12930	75.3	69.9	27.9	(0.51, 0.49)

Table S8. EL performances of various orange-red OLEDs based on TADF emitters with emission peaks from 560 to 650 nm previously reported in the literature.[3-30]

Emitter	λ_{EL} [nm]	V_{on} [V]	$\eta_{C,max}$ [cd A^{-1}]	$\eta_{P,max}$ [lm W^{-1}]	$\eta_{ext,max}$ [%]	CIE (x, y)	Ref.
**DPPM-SXAC**	**570**	**3.1**	**95.3**	**93.5**	**31.8**	**(0.48, 0.50)**	**This work**
**DPPM-SXAC**	**572**	**3.1**	**87.4**	**85.8**	**33.5**	**(0.49, 0.50)**	**This work**
**DPPM-STAC**	**574**	**3.3**	**87.4**	**80.7**	**29.3**	**(0.48, 0.51)**	**This work**
**DPPM-SFAC**	**578**	**3.1**	**82.1**	**77.0**	**30.6**	**(0.51, 0.49)**	**This work**
**DPPM-DPAC**	**574**	**3.1**	**82.5**	**81.0**	**27.6**	**(0.49, 0.51)**	**This work**
**DPPM-DMAC**	**596**	**3.1**	**47.9**	**47.0**	**23.1**	**(0.56, 0.44)**	**This work**
PXZBM	567	4.9	30.3	18.7	10.9	(0.45, 0.51)	3
1DMAC-BP	560	3.7	28.5	22.4	10.1	(0.43, 0.54)	4
2DMAC-BP	576	3.1	33.7	30.2	11.8	(0.43, 0.54)	4
3DMAC-BP	606	3.1	38.2	36.4	22.0	(0.43, 0.54)	4
PzTDBA	576	2.6	68.7	-	30.3	(0.49, 0.50)	5
PzDBA	595	2.7	35.7	-	21.8	(0.55, 0.45)	5
PXZ-PCN	568	2.6	66.9	48.3	15.1	(0.48, 0.51)	6
Bis-PXZ-PCN	600	2.9	19.5	18.2	9.8	(0.55, 0.44)	6
Tri-PXZ-PCN	608	2.9	18.3	17.9	9.7	(0.56, 0.44)	6
PXZ-DCPP	608	3.2	29.3	27.8	17.4	(0.56, 0.43)	7
Ac-CNP	580	4.7	38.1	26.1	13.3	(0.47, 0.51)	8
Px-CNP	606	5.5	5.8	3.1	3.0	(0.53, 0.44)	8
SpAcDBA	567	3.0	84.7±2.6	76.6±2.2	30.0±0.8	(0.46, 0.52)	9
dmAcDBA	583	3.2	58.3±3.4	48.6±1.9	24.9±0.5	(0.51, 0.48)	9
dPhADBA	613	3.5	15.2±1.5	12.3±1.1	11.1±0.5	(0.60, 0.39)	9
TRZ-SBA-NAI	593	2.9	71.0	79.6	31.7	(0.55. 0.45)	10
SBDBQ-PXZ	572	3.1	29.1	23.4	11.1	(0.49, 0.50)	11
DBQ-3PXZ	572	3.4	36.1	28.1	14.1	(0.50, 0.49)	11
NAI-DPAC	584	3.0	76.2	79.7	29.2	(0.52, 0.47)	12
NAI-DMAC	597	3.0	50.7	53.1	23.4	(0.56, 0.44)	12
PyCN-ACR	572	3.4	-	-	15.6	(0.46, 0.52)	13
DMAC-Ph-DCPP	596	3.3	34.5	32.8	16.9	(0.53, 0.46)	14
DPA-DCPP	616	3.0	14.4	15.1	10.4	(0.61, 0.38)	14
DMAC-DCPP	624	3.3	12.8	12.2	10.1	(0.60, 0.40)	14
DPA-Ph-DCPP	644	3.2	13.2	12.9	15.1	(0.64, 0.36)	14
DPXZ-BPPZ	612	3.1	30.3 ± 0.6	30.9 ± 1.3	20.1	(0.60, 0.40)	15

ANQDC-MeFAC	614	2.9	41.4	46.5	26.3	(0.60, 0.40)	16
ANQDC-DMAC	615	2.7	47.6	53.1	27.5	(0.58, 0.41)	16
Ac-CNBQx	617	2.8	28.9	32.4	20.0	(0.59, 0.41)	17
Ac-CNBPz	630	2.8	18.2	17.2	16.2	(0.61, 0.39)	17
HAP-3TPA	610	4.4	25.9±1.6	22.1±1.2	17.5±1.3	(0.60, 0.40)	18
PT-TPA	632	2.6	30	38.5	29.7	(0.65,0.35)	19
3PXZ-BP	634	2.5	7.8	8.8	7.1	(0.62, 0.37)	20
Pz-DMNI	635	6.0	16.8	9.3	7.1	(0.59, 0.40)	21
T-DA-2	640	3.0	24.4	22.5	26.3	(0.62, 0.37)	22
BTDMAc-NAI	641	4.0	9.6	7.3	9.2	(0.62, 0.38)	23
BTDPAc-PhNAI	642	4.0	10.1	7.6	10.1	(0.61,0.39)	24
TPA-QCN	644	3.0	14.3	14.9	14.5	(0.62, 0.38)	25
DDTPACz-DCPP	646	4.0	13.6	9.0	13.6	(0.61, 0.38)	26
TPA-DCPP	648	3.4	-	6.8	9.6	(0.64, 0.35)	27
TPA-PPDCN	648	3.2	13.9	14.0	18.8	(0.65, 0.35)	28
TPA-PZCN	648	2.4	20	26.3	28.1	(0.66, 0.34)	29
DCPPr-α-NDPA	606	3.1	59.6	58.4	31.5	(0.58, 0.42)	30

References

[1] M. J. Frisch, G. W. Trucks, H. B. Schlegel, G. E. Scuseria, M. A. Robb, J. R. Cheeseman, G. Scalmani, V. Barone, B. Mennucci, G. A. Petersson, H. Nakatsuji, M. Caricato, X. Li, H. P. Hratchian, A. F. Izmaylov, J. Bloino, G. Zheng, J. L. Sonnenberg, M. Hada, M. Ehara, K. Toyota, R. Fukuda, J. Hasegawa, M. Ishida, T. Nakajima, Y. Honda, O. Kitao, H. Nakai, T. Vreven, J. J. A. Montgomery, J. E. Peralta, F. Ogliaro, M. J. Bearpark, J. Heyd, E. N. Brothers, K. N. Kudin, V. N. Staroverov, R. Kobayashi, K. R. J. Normand, A. P. Rendell, J. C. Burant, S. S. Iyengar, J. Tomasi, M. Cossi, N. Rega, N. J. Millam, M. Klene, J. E. Knox, J. B. Cross, V. Bakken, C. Adamo, J. Jaramillo, R. Gomperts, R. E. Stratmann, O. Yazyev, A. J. Austin, R. Cammi, C. Pomelli, J. W. Ochterski, R. L. Martin, K. Morokuma, V. G. Zakrzewski, G. A. Voth, P. Salvador, J. J. Dannenberg, S. Dapprich, A. D. Daniels, O. Farkas, J. B. Foresman, J. V. Ortiz, J. Cioslowski, D. J. Fox, Gaussian, *Inc.*, Wallingford, *CT*, USA **2009**.

[2] J. Liang, C. Li, Y. Cui, Z. Li, J. Wang, Y. Wang, *J. Mater. Chem. C* **2020**, *8*, 1614.

[3] D.-G. Chen, T.-C. Lin, C.-L. Chen, Y.-T. Chen, Y.-A. Chen, G.-H. Lee, P.-T. Chou, C.-W. Liao, P.-C. Chiu, C.-H. Chang, Y.-J. Lien, Y. Chi, *ACS Appl. Mater. Interfaces* **2018**, *10*, 12886.

[4] F.-M. Xie, H.-Z. Li, G.-L. Dai, Y.-Q. Li, T. Cheng, M. Xie, J.-X. Tang, X. Zhao, *ACS*

Appl. Mater. Interfaces **2019**, *11*, 26144.

[5] D. Karthik, Y. H. Jung, H. Lee, S. Hwang, B.-M. Seo, J.-Y. Kim, C. W. Han, J. H. Kwon, *Adv. Mater.* **2021**, *33*, 2007724.

[6] Z. Chen, Z. Wu, F. Ni, C. Zhong, W. Zeng, D. Wei, K. An, D. Ma, C. Yang, *J. Mater. Chem. C* **2018**, *6*, 6543.

[7] B. Wang, X. Qiao, Z. Yang, Y. Wang, S. Liu, D. Ma, Q. Wang, *Org. Electron.* **2018**, *59*, 32.

[8] I. S. Park, S. Y. Lee, C. Adachi, T. Yasuda, *Adv. Funct. Mater.* **2016**, *26*, 1813.

[9] C.-M. Hsieh, T.-L. Wu, J. Jayakumar, Y.-C. Wang, C.-L. Ko, W.-Y. Hung, T.-C. Lin, H.-H. Wu, K.-H. Lin, C.-H. Lin, S. Hsieh, C.-H. Cheng, *ACS Appl. Mater. Interfaces* **2020**, *12*, 23199.

[10] X. Zeng, Y.-H. Huang, S. Gong, P. Li, W.-K. Lee, X. Xiao, Y. Zhang, C. Zhong, C.-C. Wu, C. Yang, *Mater. Horiz.* **2021**, *8*, 2286.

[11] L. Yu, Z. Wu, G. Xie, W. Zeng, D. Ma, C. Yang, *Chem. Sci.* **2018**, *9*, 1385.

[12] W. Zeng, H.-Y. Lai, W.-K. Lee, M. Jiao, Y.-J. Shiu, C. Zhong, S. Gong, T. Zhou, G. Xie, M. Sarma, K.-T. Wong, C.-C. Wu, C. Yang, *Adv. Mater.* **2018**, *30*, 1704961.

[13] X. Cai, X. Li, G. Xie, Z. He, K. Gao, K. Liu, D. Chen, Y. Cao, S.-J. Su, *Chem. Sci.* **2016**, *7*, 4264.

[14] S. Wang, Z. Cheng, X. Song, X. Yan, K. Ye, Y. Liu, G. Yang, Y. Wang, *ACS Appl. Mater. Interfaces* **2017**, *9*, 9892.

[15] J.-X. Chen, K. Wang, C.-J. Zheng, M. Zhang, Y.-Z. Shi, S.-L. Tao, H. Lin, W. Liu, W.-W. Tao, X.-M. Ou, X.-H. Zhang, *Adv. Sci.* **2018**, *5*, 1800436.

[16] X. Gong, P. Li, Y.-H. Huang, C.-Y. Wang, C.-H. Lu, W.-K. Lee, C. Zhong, Z. Chen, W. Ning, C.-C. Wu, S. Gong, C. Yang, *Adv. Funct. Mater.* **2020**, *30*, 1908839.

[17] R. Furue, K. Matsuo, Y. Ashikari, H. Ooka, N. Amanokura, T. Yasuda, *Adv. Opt. Mater.* **2018**, *6*, 1701147.

[18] J. Li, T. Nakagawa, J. MacDonald, Q. Zhang, H. Nomura, H. Miyazaki, C. Adachi, *Adv. Mater.* **2013**, *25*, 3319.

[19] Y.-Y. Wang, K.-N. Tong, K. Zhang, C.-H. Lu, X. Chen, J.-X. Liang, C.-K. Wang, C.-C. Wu, M.-K. Fung, J. Fan, *Mater. Horiz.* **2021**, *8*, 1297.

[20] F.-M. Xie, P. Wu, S.-J. Zou, Y.-Q. Li, T. Cheng, M. Xie, J.-X. Tang, X. Zhao, *Adv. Electron. Mater.* **2020**, *6*, 1900843.

[21] Y. Wu, X. Chen, Y. Mu, Z. Yang, Z. Mao, J. Zhao, Z. Yang, Y. Zhang, Z. Chi, *Dyes Pigm.* **2019**, *169*, 81.

[22] Y T. Yang, Z. Cheng, Z. Li, J. Liang, Y. Xu, C. Li, Y. Wang, *Adv. Funct. Mater.* **2020**, *30*, 2002681.

[23] T. Chen, C.-H. Lu, C.-W. Huang, X. Zeng, J. Gao, Z. Chen, Y. Xiang, W. Zeng, Z. Huang, S. Gong, C.-C. Wu, C. Yang, *J. Mater. Chem. C* **2019**, *7*, 9087.

[24] T. Chen, C.-H. Lu, Z. Chen, X. Gong, C.-C. Wu, C. Yang, *Chem. Eur. J.* **2021**, *27*, 3151.

[25] C. Li, R. Duan, B. Liang, G. Han, S. Wang, K. Ye, Y. Liu, Y. Yi, Y. Wang, *Angew. Chem. Int. Ed.* **2017**, *56*, 11525.

[26] B. Wang, H. Yang, Y. Zhang, G. Xie, H. Ran, T. Wang, Q. Fu, Y. Ren, N. Sun, G. Zhao, J.-Y. Hu, Q. Wang, *J. Mater. Chem. C* **2019**, *7*, 12321.

[27] S. Wang, X. Yan, Z. Cheng, H. Zhang, Y. Liu, Y. Wang, *Angew. Chem. Int. Ed.* **2015**, *54*, 13068.

[28] T. Yang, B. Liang, Z. Cheng, C. Li, G. Lu, Y. Wang, *J. Phys. Chem. C* **2019**, *123*, 18585.

[29] Y.-L. Zhang, Q. Ran, Q. Wang, Y. Liu, C. Hanisch, S. Reineke, J. Fan, L.-S. Liao, *Adv. Mater.* **2019**, *31*, 1902368.

[30] Z. Cai, X. Wu, H. Liu, J. Guo, D. Yang, D. Ma, Z. Zhao, B. Z. Tang, *Angew. Chem. Int. Ed.* **2021**, *60*, 23635.
